# Whole-genome sequencing revealed the genomic origin of *Solanum demissum* Lindl., a Mexican hexaploid wild potato species

**DOI:** 10.1186/s12870-026-08972-2

**Published:** 2026-05-22

**Authors:** Awie J. Hosaka, Kazuyoshi Hosaka

**Affiliations:** 1 Nihon BioData Corporation, Takatsu, Kawasaki, Kanagawa 213-0033 Japan; 2 Present address: Ac-Planta Inc., 75-1 Onocho, Tsurumi, Yokohama, Kanagawa 230-0046 Japan; 3 Kihara Institute for Biological Research, Yokohama City University, Yokohama, 244-0813 Japan; 4 Potato Germplasm Enhancement Laboratory, Obihiro University of Agriculture and Veterinary Medicine, Obihiro, Hokkaido 080-8555 Japan

**Keywords:** Potato, *Solanum demissum*, Hexaploid, Mexican wild potatoes, Chromosome-scale assembly, Crop wild relatives, *Solanum acaule*, *Solanum verrucosum*

## Abstract

**Background:**

*Solanum demissum* Lindl. (2*n* = 6*x* = 72) is the most popular wild species in potato breeding because of its resistance to late blight, caused by *Phytophthora infestans* (Mont.) de Bary. Regular meiosis with 36 bivalents indicates that *S. demissum* has three distinct genomes, but their origin remains unknown.

**Results:**

We constructed a chromosome-scale assembly of *S. demissum* using PacBio long-read sequencing and Hi-C scaffolding technologies. The final assembly comprised 2.035 Gb anchored to 36 chromosome-level scaffolds, which were phased into three subgenomes (SG1, SG2, and SG3, with sizes of 696.51 Mb, 685.32 Mb, and 653.29 Mb, respectively). Evidence-based annotation, using RNA-seq datasets, and deep learning-based prediction identified 32,888 genes in SG1, 33,028 in SG2, and 31,999 in SG3. Comparing gene sequences among the three subgenomes and 47 other genomes from diverse collections distinctly placed these subgenomes in the phylogenetic tree; the most closely related were SG1 with the maternal and A-genome ancestor *S. verrucosum*, SG2 with SG2 of *S. acaule*, and SG3 with SG1 of *S. acaule*. Syntenic ratios, indicating genome structure similarity, further supported these relationships. The three subgenomes of *S. demissum* exhibited mostly balanced gene expression. Additionally, the composition and genome occupancy of transposable elements, as well as the number of resistance genes, are similar across the three subgenomes and more closely resemble those of the ancestral genomes in *S. acaule* and *S. verrucosum*.

**Conclusions:**

*Solanum demissum* likely originated between a Mexican diploid *S. verrucosum* as the female parent and a South American tetraploid *S. acaule* as the male parent.

**Supplementary Information:**

The online version contains supplementary material available at 10.1186/s12870-026-08972-2.

## Introduction

The common potato (*Solanum tuberosum* L., 2*n* = 4*x* = 48) is the fourth most important food crop grown worldwide [26]. It has an enormous genetic reservoir, comprising over 100 closely related wild species from section *Petota* of the genus *Solanum* [38, 123]. These wild species vary in ploidy from diploid to hexaploid and are distributed from the central United States through Central America to Chile, with the Mexican highlands and the central Andes as centers of diversity [43]. Hawkes [38] recognized over 200 wild species and separated non-tuber-bearing and tuber-bearing species in section *Petota* into the subsections *Estolonifera* and *Potatoe*, respectively, and the species in subsection *Potatoe* into 19 taxonomic series. Recent molecular phylogenies have supported these two subsections as the Etuberosum and Petota clades, respectively, and together with Tomato and some other minor clades, they form the large Potato clade [29]. According to chloroplast DNA restriction site data, section *Petota* consists of four clades: Clade 1 contains the North and Central American diploid species, exclusive of *S. bulbocastanum* Dun., *S. cardiophyllum* Lindl., and *S. verrucosum* Schltdl.; Clade 2 contains *S. bulbocastanum* and *S. cardiophyllum*; Clade 3 contains all examined members of the South American series *Piurana* and some South American species placed in other series; Clade 4 contains all remaining South American species, *S. verrucosum*, and the North and Central American polyploid species, including all cultivated potato species [117]. Later, Clade 1 and Clade 2 were combined, and the three-clade phylogeny has been supported by full chloroplast DNA sequence data [53, 138], DNA sequences of granule-bound starch synthase I [122], nitrate reductase [108], and conserved orthologs [3], genome-wide single-nucleotide polymorphism (SNP) analysis [76], and whole-genome sequencing [1, 131, 148]. Among the 19 taxonomic series, series *Acaulia*, *Longipedicellata*, and *Demissa* consist exclusively of polyploid species. Well-known species in each series are *S. acaule* Bitter (2*n* = 4*x* = 48), *S. stoloniferum* Schltdl. (2*n* = 4*x* = 48), and *S. demissum* Lindl. (2*n* = 6*x* = 72), respectively. Compared to diploid species, which are usually adapted to narrow niches, *S. stoloniferum* and *S. demissum* are widely distributed in the Mexican highlands and adjacent regions. *Solanum acaule* is the most widely distributed species, extending from Ecuador southwards into northern Argentina [38, 124].

Genome similarity and differentiation are essential for understanding and utilizing genetic diversity as a genetic resource in breeding. Traditionally, genome similarity has been evaluated based on chromosome-pairing behaviors during meiosis. Diploid hybrids between South American species usually exhibit regular meiosis with 12 bivalents, indicating that the common A genome is shared among them [51]. *Solanum tuberosum* forms 2–4 multivalents (trivalents and quadrivalents); thus, it is cytogenetically a segmental allotetraploid (genome formula AAA^t^A^t^; [85]), although it is generally referred to as an autotetraploid (genomic formula AAAA). In contrast, *S. acaule*, *S. stoloniferum*, and *S. demissum* show regular meiosis with complete formation of bivalents [85]. Triploid hybrids, generated from a diploid A-genome species with *S. acaule* exhibit a high frequency of trivalents, indicating that the same A genome is shared [130]. A dihaploid clone induced through anther culture from *S. acaule* presented 10.3 to 10.6 bivalents and 2.8 to 3.4 univalents [13, 137], indicating considerable similarity between the two genomes of *S. acaule*. Thus, the genome formula AAA^a^A^a^ has been given to *S. acaule* [83]. In contrast, triploid hybrids between *S. stoloniferum* and a diploid A-genome species presented 12 bivalents and 12 univalents, indicating that *S. stoloniferum* has an A and another genome [80, 81]. A dihaploid clone of *S. stoloniferum* predominantly exhibited univalents at meiosis, supporting its allotetraploid nature [56]. The second genome of *S. stoloniferum* has been referred to as the B genome [85]. Whole-genome sequencing of *S. stoloniferum* revealed its allopolyploid nature, and its B genome is highly homologous to the genomes of the Mexican diploid species belonging to Clade 1+2 [48]. The other genome so far identified in the diploid species is the E genome in the non-tuber-bearing species in the series *Etuberosa* [104].

*Solanum demissum* is the most popular wild potato species since the early 1900s because of its resistance to late blight, caused by *Phytophthora infestans* (Mont.) de Bary [101, 109]. *Solanum demissum* is highly self-fertile and sets abundant berries in nature. When *S. demissum* is used as a female parent, pentaploid hybrids (2*n* = 60) can be easily obtained from a cross with *S. tuberosum* [20, 111]. By continued backcrossing, many late blight resistant cultivars were released [101, 109]. To date, 11 race-specific resistance genes *R1* − *R11* have been identified from *S. demissum*, of which *R1*, *R2*, *R3a*, *R3b*, and *R8* have been cloned [7, 55, 72, 78, 134]. However, the efficient reproduction of *P. infestans* in both asexual and sexual forms, combined with fast-evolving effector *R* genes necessary for successful infection [34], makes it a rapidly evolving pathogen that can readily generate new virulent strains. Consequently, none of the single or stacked resistance genes, even when combined in a single cultivar, conferred durable resistance [28]. The rapid adaptation of *P. infestans* makes it very difficult to breed durably resistant potato varieties [28, 87].

*Solanum demissum* regularly forms 36 bivalents at meiosis [79, 130]. Different clones of its triploid haploid formed 4.74 to 9.78 bivalents per cell in addition to univalents [5, 22, 52, 56, 79]. These observations suggested that *S. demissum* has two somewhat similar genomes and a third genome that is rather different. Mean bivalents frequencies in the tetraploid hybrids of *S. demissum* with A-genome diploid species have been reported as follows: 17.64 and 21.21 with *S. verrucosum* ( [102] and [65], respectively), 16.25−-16.46 with *S. chacoense*, 19.78 with *S. phureja* Juz. et Buk., and 20.47 with *S. stenotomum* Juz. et Buk. [82]. Based on these observations, the third genome of *S. demissum* is considered to be an A genome [56, 79, 82]. The currently proposed genome formula is AADDD^d^D^d^ [85]. The most likely maternal and A-genome ancestor is *S. verrucosum*, as suggested by chloroplast DNA similarity and the shared nature of a mitochondrial molecule called Band 1 [49, 110, 119].

However, genomic in situ hybridization (GISH) analysis revealed that *S. demissum* has all three chromosome sets related to the basic A genome, similar to the GISH results for *S. acaule* [98]. Granule-bound starch synthase and nitrate reductase gene phylogenies indicated that the two distinct alleles identified in *S. demissum* clustered within the same clade as A-genome species [108, 122]. Molecular marker analyses revealed that the species most closely related to *S. demissum* are *S. acaule* and *S. albicans* (Ochoa) Ochoa of the series *Acaulia* [19, 63, 91]. *Solanum albicans* (2*n* = 6*x* = 72) was likely derived from *S. acaule* and a diploid species from another series [37], which possesses a clearly differentiated genome from A or A^a^ genomes [44, 85]. Many authors have recognized morphological similarities among *S. demissum*, *S. acaule*, and *S. albicans* [17, 39, 40, 62, 94, 120]. Along with molecular similarities, Spooner et al. [121] grouped *S. demissum*, *S. acaule*, and *S. albicans* into a single group, the Acaulia Group. Recently, Achakkagari et al. [1] conducted whole-genome sequencing for *S. acaule* and revealed that it originated from a maternal progenitor *S. megistacrolobum* Bitt. (combined into *S. boliviense* Dun. by Spooner et al. [124], an A-genome species from Clade 4, and an unknown or extinct species from Clade 3. Clade 3 is a large clade containing approximately one-third of the tuber-bearing *Solanum* species and is relatively unexploited [3, 123]. Thus, the second genome of *S. acaule* likely originated from an unexamined species of Clade 3.

The presence and source of the distinct genome D or D^d^ in *S. demissum* have remained unknown. In this study, we sequenced the three subgenomes of *S. demissum* and revealed that the most closely related genomes were the A genome of *S. verrucosum* and the two *S. acaule* subgenomes. Therefore, we suggest that *S. demissum* originated from *S. verrucosum* as the female and *S. acaule* as the male. Potential disease- and pest-resistance genes were identified in the *S. demissum* genome, and their significance and aspects for utilization are also discussed.

## Materials and methods

### Plant material

*Solanum demissum* clone 24H65-12 was used for sequencing, which was derived by self-crossing from 5H109-5. 5H109-5 was initially grown as a seedling (accession PI 186551), obtained from the US Potato Genebank at Sturgeon Bay, Wisconsin, USA, and used for genetic map construction [95] and QTL analysis of crossability [45].

### Sequencing

Young leaves were collected from the mature plant and frozen until use. Large-sized DNA was extracted from frozen leaves using a NucleoBond HMW DNA kit (Macherey-Nagel, Düren, Germany) according to the manufacturer’s protocol, with modifications as previously described [48]. PacBio HiFi reads were commercially obtained from the *S. demissum* genomic DNA at Macrogen Japan Corp. (Tokyo, Japan) using the PacBio Revio (PacBio). The Hi-C library was prepared as previously described [48]. The NextSeq 1000 (Illumina, Inc.) was used for sequencing the Hi-C library according to the manufacturer’s protocol.

### Genome assembly

Hifiasm 0.25.0-r726 [15] was used for assembly with HiFi reads, employing the “–hom-cov 93” option based on the sequence coverage. To remove organellar genome-derived sequences from the assembled contigs, contigs shorter than 1 Mb were first extracted. Homologous regions to the mitochondrial and chloroplast genomes of *S. phureja* (DM v 8.1; Yang et al. [141] were subsequently searched using blastn 2.12.0 [2]. Contigs containing homologous regions longer than 10 kb were considered organellar-derived and excluded from downstream analyses. For chromosome-level assembly, a Hi-C library was prepared and sequenced on the NextSeq 1000. Hi-C reads were aligned to the contigs using BWA 0.7.17-r1188 [74]. PCR duplicates were removed using SAMBLASTER [27], and secondary and supplementary alignments were filtered out using the SAMtools view command with the “samtools view -S -h -b -F 3340” option [75]. The resulting BAM file was used in the HapHiC 1.0.7 pipeline [145] to scaffold contigs onto chromosomes. During this step, the option “-nchr 36” was specified to indicate the expected number of chromosomes. The scaffolds were reviewed and manually corrected using JuiceBox 1.11.08 (https://github.com/aidenlab/Juicebox). To phase the chromosomes into three subgenomes, SubPhaser 1.2.6. [58] was used with default parameters.

### RNA-seq

RNA was extracted from leaves, floral buds, flowers, and stolons of the mature *S. demissum* plant using a Plant/Fungi Total RNA Purification Kit (Norgen Biotek Corp., Ontario, Canada). After fresh samples were frozen in liquid nitrogen, ground in a mortar with a pestle, and lysed with 600 µl of Lysis Buffer C at 55 ℃ for 5 min, the lysate was centrifuged at 12,000 rpm for 2 min to precipitate large debris and applied to the Filter Column. Subsequent steps were followed according to the manufacturer’s protocol, including DNase treatment with RNase-Free DNase I Kit (Norgen Biotek Corp.). Three biological replicates were taken from each tissue. Library preparation and sequencing were performed commercially by Novogene (Nippon Genetics Co., Ltd., Tokyo, Japan). The raw reads were qualified and trimmed with fastp 0.23.2 [16].

### Annotation of protein-coding regions

For annotation of the *S. demissum* genome, RNA-seq datasets were initially utilized for evidence-based annotation, as previously described [48]. Next, deep-learning-based annotation was performed by using the Helixer v0.3.4 web tool [127]. To merge the evidence-based annotation and deep-learning-based annotation, GffCompare v0.12.10 was used with the Helixer GFF as the query and the StringTie/TransDecoder-derived GFF3 as the reference (-r), with outputs written under the prefix dem_gffcmp (-o) [68, 99]. From the resulting .tmap file, entries assigned the class code u (transcripts unknown with respect to the reference, i.e., intergenic/novel) were extracted, and the corresponding query transcript IDs were collected. The original Helixer GFF was then filtered to retain only these u-class features, ensuring that only putative novel Helixer models were preserved while overlapping predictions were excluded. The retained Helixer features were concatenated with the StringTie/TransDecoder-derived GFF3, and the combined records were lexicographically sorted by sequence ID to generate a unified GFF file. To standardize feature identifiers, AGAT v1.3.3 (https://zenodo.org/records/18230424) was applied with a project-specific prefix (–prefix dem) and TAIR-style naming (–tair), resulting in a final annotation dataset.

### Evaluation of assembly and annotation quality

Statistics on contig count and length distribution for both the draft and final assemblies were generated using the “stat” function in Seqkit v0.15.0 [113]. The completeness of genome assembly and gene annotation was further evaluated using Benchmarking Universal Single-Copy Orthologs (BUSCO; [114]), employing the Solanales odb10 dataset, which provides genomic and protein sequence references [69].

### TE annotation

Annotation of transposable elements (TEs) and phylogenetic analyses within *S. demissum* and related species were conducted as previously described [48]. Briefly, TEs were identified using EDTA v2.0 [96]. The two long terminal repeats (LTRs) at both ends of a retrotransposon are identical at the beginning of the insertion, but their differences increase over time. Thus, we obtained LTR identity information from intact TE annotations and examined LTR identity distributions as a proxy for insertion age to assess recent TE activity. To infer transposition histories of TEs, sequence divergence of TEs relative to their consensus sequences was estimated using RepeatMasker v4.1.1 (Smit AFA, Hubley R, and Green P, at http://repeatmasker.org) with EDTA-generated TE libraries, followed by calculating Kimura two-parameter (K2P) divergence metrics [67] using the accompanying RepeatMasker utility scripts. For Gypsy retrotransposon analyses, intact elements were extracted and classified based on conserved protein domains using TEsorter [146] with the REXdb-plant database [92]. Phylogenetic relationships were inferred from concatenated reverse transcriptase (RT), RNase H (RH), and integrase (INT) domains using VeryFastTree 4.0.3 [100], and trees were visualized with GGTREE 3.10.1 [143].

### HiFi read mapping, variant calling, and window-based heterozygosity analysis

PacBio HiFi reads were aligned to the *S. demissum* reference genome (subgenome-resolved scaffolds) using minimap2 [73] with the map-hifi preset. The resulting alignments were converted to BAM format, sorted, and indexed using SAMtools 1.61.1 [75]. Single-nucleotide variants were called from the mapped reads using BCFtools 1.19 [18]. Briefly, pileup files were generated with the “bcftools mpileup” command, and variants were called with the “bcftools call” command under a diploid model. Variant calls were output in VCF format for downstream analyses. To assess genome-wide patterns of read depth and heterozygosity, the reference genome was divided into 1-Mb non-overlapping windows using BEDTools [103]. Read depth for each window was calculated using Mosdepth 0.3.3 [97]. Variant calls were filtered based on read depth to retain high-confidence sites. Only variants with total read depth values between 50 and 200, as estimated from both the DP and DP4 fields in the VCF, were retained. From these filtered variants, heterozygous sites (0/1 genotypes) were extracted. The number of heterozygous variants within each 1-Mb window was then counted using BEDTools, generating a chromosome-scale profile of heterozygosity across the genome.

### Expression analysis

RNA-seq reads were aligned to the *S. demissum* genome using HISAT2 v2.2.1 [66]. Then, read counts for each gene were calculated using featureCounts v2.0.6 [77] and normalized to transcripts per million (TPM). Spearman’s rank correlation of single-copy orthologs was calculated from the TPM-normalized expression matrix, and a distance matrix was used for hierarchical clustering with the Ward.D2 method [90]. The clustering results were visualized as a dendrogram. To assess expression balance among subgenomes, only ortholog sets in which each gene was assigned to all three subgenomes (SG1, SG2, and SG3) were used. For each ortholog set, a proportion of the TPM value in each homoeolog to the sum of TPM values of the three homoeologs was calculated. The resulting expression balances were visualized using the R package ggtern [35]. For descriptive classification of homoeolog expression bias, ortholog sets were categorized as SG1-, SG2-, or SG3-dominant when one subgenome accounted for more than 60% of the total expression, and each of the other two accounted for less than 20%. Ortholog sets were categorized as SG1-, SG2-, or SG3-suppressed when one subgenome accounted for less than 20% of the total expression, and each of the other two accounted for more than 20%. All remaining ortholog sets were classified as balanced.

### Phylogenetic inference

The phylogenetic relationships of the subgenomes of *S. demissum* among tuber-bearing *Solanum* species were inferred using OrthoFinder [25], as previously described [48]. The dataset comprised 50 genomes listed in Table 1, including a broad representation of diploid tuber-bearing *Solanum* species, the *S. demissum* subgenomes (SG1, SG2, and SG3), the subgenomes of the allotetraploids *S. stoloniferum* (A and B) and *S. acaule* (SG1 and SG2), and eggplant (*S. melongena*) as an outgroup (Table 1). Most of the whole-genome sequence data were obtained from publicly available databases. Briefly, single-copy ortholog groups were detected and aligned using MAFFT [64] by enabling the “-M msa” and “-A mafft” options. The tree was inferred using IQ-TREE 2 [89] with the “-m MFP” and “-bb 1000” options. The tree was visualized using FigTree v1.4.4 (http://tree.bio.ed.ac.uk/software/figtree/).

**Table 1:** *Solanum* genomes compared in this study

Hawkes’ taxonomic series and species^a)^	Accession	Spooner’s taxonomy	Genome designation in this study^b)^	Reference
*S. melongena* (2*x*)	67/3	*S. melongena*	*S. melongena* v4.0*	Barchi et al. [8]
*S. lycopersicum* (2*x*)	cv. Heinz 1706	*S. lycopersicum*	*S. lycopersicum* v5.0*	Zhou et al. [149]
Series* Etuberosa*				
*S. brevidens* (2*x*; A, Ch)	PG0009 (PI 558254)	*S. palustre*	*S. palustre*	Tang et al. [131]
*S. etuberosum* (2*x*; Ch)	PG0019 (PI 558302)	*S. etuberosum*	S. etuberosum*	Tang et al. [131]
Series* Morelliformia*				
* S. morelliforme* (2*x*; G, M, H)	PG1011 (PI 243357)	*S. morelliforme*	*S. morelliforme*	Tang et al. [131]
Series* Bulbocastana*				
* S. bulbocastanum* (2*x*; G, H, M)	11H21 (PI 666967)	*S. bulbocastanum*	S. bulbocastanum*	Hosaka et al. [47]
Series* Pinnatisecta*				
*S. brachistotrichum* (2*x*; M)	PG1015 (PI 498216)	*S. stenophyllidium*	S. stenophyllidium*	Zhang et al. [148]
*S. cardiophyllum* ssp. *cardiophyllum* (2*x*; M)	23H17-1 (PI 283062)	*S. cardiophyllum*	*S. cardiophyllum*	Hosaka et al. [48]
*S. cardiophyllum* ssp*. ehrenbergii* (2*x*; M)	PG1006 (PI 275212)	*S. ehrenbergii*	S. ehrenbergii*	Zhang et al. [148]
*S. jamesii* (2*x*; M, USA)	PG1008 (PI 458423)	*S. jamesii*	*S. jamesii*	Tang et al. [131]
*S.* × *michoacanum* (2*x*; M)	PG1023 (PI 653799)	*S. michoacanum*	S. michoacanum*	Zhang et al. [148]
*S. pinnatisectum* (2*x*; M)	PG1013 (PI 275232)	*S. pinnatisectum*	*S. pinnatisectum*	Tang et al. [131]
*S. tarnii* (2*x*; M)	PG1018 (PI 570641)	*S. tarnii*	S. tarnii*	Zhang et al. [148]
Series* Commersoniana*				
*S. commersonii* ssp. *commersonii* (2*x*; A, Br, U)	PG4049(PI 473412)	*S. commersonii*	*S. commersonii*	Tang et al. [131]
*S. commersonii* ssp*. malmeanum* (2*x*; A, Br, Pr, U)	PG4019 (PI 472841)	*S. malmeanum*	S. malmeanum*	Zhang et al. [148]
Series* Circaeifolia*				
* S. circaeifolium* (2*x*; B)	PG4033 (PI 498117)	*S. stipuloideum*	S. stipuloideum*	Zhang et al. [148]
Series* Lignicaulia*				
* S. lignicaule* (2*x*; P)	PG4017 (PI 473351)	*S. lignicaule*	*S. lignicaule*	Tang et al. [131]
Series* Yungasensa*				
*S. chacoense* (2*x*; A, B, Br, Pr, P, U)	M6	*S. chacoense*	*S. chacoense* M6 v5*	https://spuddb.uga.edu/M6_v5_0_download.shtml
*S. huancabambense* (2*x*; P)	PG3008 (PI 498244)	*S. huancabambense*	S. huancabambense*	Zhang et al. [148]
Series* Megistacroloba*				
*S. megistacrolobum* (2*x*; A, B, P)	PG5076 (PI 473122)	*S. boliviense*	*S. boliviense*	Tang et al. [131]
*S. sogarandinum* (2*x*; P)	PG4032 (PI 365360)	*S. sogarandinum*	*S. sogarandinum*	Tang et al. [131]
Series* Cuneoalata*				
* S. infundibuliforme* (2*x*; A, B)	PG4013 (PI 597699)	*S. infundibuliforme*	S. infundibuliforme*	Zhang et al. [148]
Series* Conicibaccata*				
*S. buesii* (2*x*; P)	PG4041 (PI 607889)	*S. buesii*	*S. buesii*	Tang et al. [131]
*S. chomatophilum* (2*x*; E, P)	PG3005 (PI 310943)	*S. chomatophilum*	*S. chomatophilum*	Tang et al. [131]
*S. irosinum* (2*x*; P)	PG4005 (PI 568985)	*S. burkartii*	*S. burkartii*	Tang et al. [131]
Series* Piurana*				
*S. acroglossum* (2*x*; P)	PG3001 (PI 498204)	*S. acroglossum*	S. acroglossum*	Zhang et al. [148]
*S. albornozii* (2*x*; E)	PG3002 (PI 561636)	*S. albornozii*	S. albornozii*	Zhang et al. [148]
*S. paucissectum* (2*x*; P)	PG3022(PI 365340)	*S. paucissectum*	*S. paucissectum*	Tang et al. [131]
*S. piurae* (2*x*; P)	PG3023 (PI 365365)	*S. piurae*	*S. piurae*	Tang et al. [131]
Series* Tuberosa*				
*S. acroscopicum* (2*x*; P)	PG4001 (PI 365314)	*S. acroscopicum*	S. acroscopicum*	Zhang et al. [148]
*S. andreanum* (2*x*; C, E)	PG3003 (PI 498148)	*S. andreanum*	*S. andreanum*	Tang et al. [131]
*S. bukasovii* (2*x*; P)	PG5068 (PI 458379)	*S. candolleanum*	S. bukasovii*	Tang et al. [131]
*S. cajamarquense* (2*x*; P)	PG6242 (CIP 762608)	*S. cajamarquense*	*S. cajamarquense*	Tang et al. [131]
*S. gourlayi* (2*x*; A, B)	PG5032(PI 545977)	*S. brevicaule*	*S. gourlayi*	Tang et al. [131]
*S. immite* (2*x*; P)	PG3009 (PI 498245)	*S. immite*	S. immite*	Zhang et al. [148]
*S. mochiquense* (2*x*; P)	PG3010 (PI 498411)	*S. mochiquense*	S. mochiquense*	Zhang et al. [148]
*S. multiinterruptum* (2*x*; P)	PG4060 (PI 498265)	*S. multiinterruptum*	*S. multiinterruptum*	Tang et al. [131]
*S. neorossii* (2*x*; A)	PG6243(CIP 764170)	*S. neorossii*	*S. neorossii*	Tang et al. [131]
*S. vernei* (2*x*; A)	PG4036(PI 473304)	*S. vernei*	*S. vernei*	Tang et al. [131]
*S. verrucosum* (2*x*; M)	11H23 (PI 666968)	*S. verrucosum*	S. verrucosum*	Hosaka et al. [46]
Series *Tuberosa* (cultivated)				
*S. phureja* (2*x*; B, C, E, P, V)	DM1-3 516 R44	*S. tuberosum* Andigenum Group (2*x*)	*S. phureja* DM8.1*	Yang et al. [141]
*S. stenotomum* ssp. *goniocalyx* (2*x*; P)	PG6359 (CIP 705468)	*S. tuberosum* Andigenum Group (2*x*)	S. stenotomum*	Tang et al. [131]
2*x* *S. tuberosum* (2*x*)	RH89-039–16	2*x* *S. tuberosum*	*S. tuberosum* RH*	Tang et al. [131]
Series* Acaulia*				
* S. acaule* (4*x*; A, B, E, P)	AA9 (PI 498202)	*S. acaule*	*S. acaule* SG1*	Achakkagari et al. [1]
			*S. acaule* SG2*	Achakkagari et al. [1]
Series* Longipedicellata*				
* S. stoloniferum* (4*x*; M, USA)	11H31	*S. stoloniferum*	*S. stoloniferum* A*	Hosaka et al. [48]
			*S. stoloniferum* B*	Hosaka et al. [48]
Series* Demissa*				
* S. demissum* (6*x*; G, M)	24H65-12 (PI 186551)	*S. demissum*	*S. demissum* SG1*	This study
			*S. demissum* SG2*	This study
			*S. demissum* SG3*	This study

^a)^Ploidy and the countries distributed are given in parentheses. Country codes are A Argentina, B Bolivia, Br Brazil, C Colombia, Ch Chile, E Ecuador, G Guatemala, H Honduras, M Mexico, Pr Paraguay, P Peru, USA United States of America, U Uruguay, and V Venezuela

^b)^Genomes and subgenomes (SGs) with chromosome-scale assemblies are shown with asterisks

### Comparison of genome structures

Syntenic regions among *S. demissum*, *S. verrucosum*, and *S. acaule* were identified using SyRI v1.6.3 [33] as previously described [48]. Centromeric regions in *S. verrucosum* were previously determined using CENH3 ChIP-seq signals [46]. The results were visualized by Plotsr 0.5.4 [32]. To further investigate genome structure similarity among chromosome-scale assemblies of *Solanum* species (shown by asterisks in Table 1), pairwise ratios of syntenic regions were calculated by dividing the length of syntenic areas by the total chromosome length of each species. Based on the ratios, a dendrogram was constructed using the unweighted pair group method with arithmetic mean (UPGMA) method, and 100 bootstrap resamplings were performed using DendroUPGMA (http://genomes.urv.cat/UPGMA/).

### Divergence analysis

To compare the sequence divergence among *S. demissum*, *S. acaule*, and *S. verrucosum*, coding DNA sequences (CDSs) in each single-copy ortholog group, detected using OrthoFinder, were translated with EMBOSS transeq v6.6.0.0 [9], aligned at the protein level using MAFFT with the “–localpair –maxiterate 1000” option, and back-translated to codon-preserving nucleotide alignments using PAL2NAL v14 [128] with the “-output paml -nogap” option. Pairwise synonymous substitution (dS) rates were estimated using codeml v4.10.7 [139], with a control file configured as follows: seqtype = 1 (codons), CodonFreq = 2 (F3 × 4), model = 2 (two or more branch ω ratios), NSsites = 0 (no among-site variation), cleandata = 1 (gap/ambiguous sites removed), and runmode = -2 (pairwise). To visualize chromosome-level patterns of synonymous divergence, dS rates were plotted along each chromosome using a sliding-window approach. For each chromosome, genes were ordered by genomic position, and the mean dS rate was calculated for consecutive windows of 10 genes.

### Detection of *R*-gene candidates

Resistance (*R*) genes are often composed of specific domains: *Drosophila* Toll and mammalian interleukin-1 receptor (TIR), coiled-coil (CC), nucleotide-binding adaptor shared by the human apoptotic protease-activating factor 1 (NB-ARC), and leucine-rich repeat (LRR) domains [107]. Nucleotide-binding and leucine-rich repeat (NLR) genes were identified in *S. demissum* and other *Solanum* species using NLR-annotator v2.1 [126] with default parameters. The chromosomal distribution of NLR genes in *S. demissum* was visualized using R 4.3.3 (https://www.r-project.org/).

## Results

### Genome assembly

**Fig. 1:**
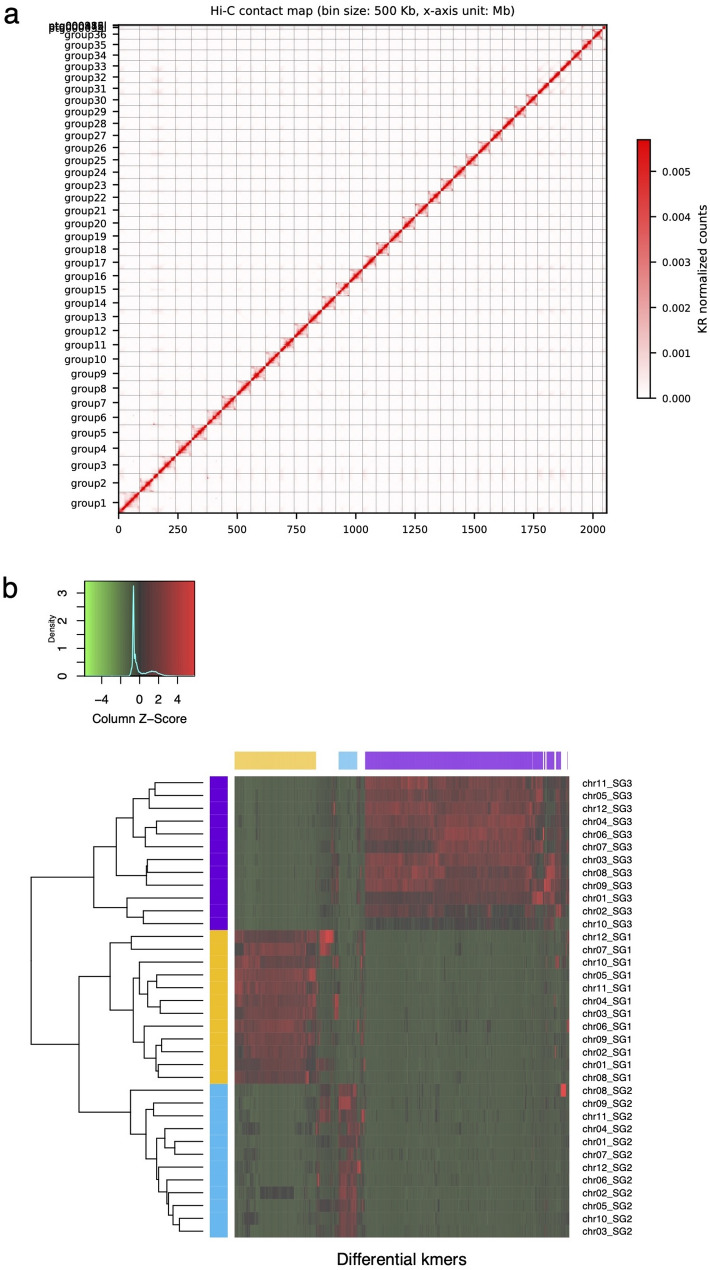
Genome assembly and subgenome phasing of *S. demissum*. **a** Hi-C contact map of *S. demissum* chromosomes. Color intensity indicates contact frequency. **b** Subgenome-specific *k*-mer distribution, grouping to three sets of 12 chromosomes

The genome of the *S. demissum* clone 24H65-12 was assembled using PacBio HiFi reads. Sequencing on the PacBio Revio generated 11,553,623 HiFi reads, yielding 211.8 Gbp of sequence data, with a mean read length of 18,334 bp and an N50 length of 18,893 bp. The initial contig assembly yielded a total length of approximately 2.517 Gb with a contig N50 of 34.07 Mb (Table 2). The 944,446,258 Hi-C read pairs (95.4 Gbp) were mapped to the contigs, processed, and scaffolded (Fig. 1a). The final nuclear genome assembly spanned 2.314 Gb, with 2.035 Gb anchored to 36 chromosome-level scaffolds. The chromosome-level scaffolding significantly improved the contiguity, achieving a scaffold N50 of 56.05 Mb (Table 2). The 36 chromosome scaffolds were phased into three subgenomes (SG1, SG2, and SG3). The *k*-mer classification heatmap confirmed the successful differentiation of the three subgenomes (Fig. 1b). The resulting subgenomes (12 chromosomes each) showed slight size asymmetry. SG1 had the largest total length, 696.51 Mb, followed by SG2, 685.32 Mb, and SG3, 653.29 Mb (Table S1).

**Table 2:** Assembly statistics of the *S. demissum* genome

Category	Number of sequences	Total length (bp)	Average length (bp)	Maximum length (bp)	N50 (bp)
Primary contig	7,456	2,516,682,040	337,537.8	78,494,623	34,073,135
Final assembly					
Chromosome	36	2,035,124,518	56,531,236.6	86,927,263	56,046,848
Unanchored	4,120	278,497,681	67,596.5	2,447,828	73,347
Organella	3,250	203,065,841	62,481.8	511,465	62,834
Chloroplast	3,060	193,913,835	63,370.5	511,465	64,396
Mitochondria	190	9,152,006	48,168.5	117,256	48,538

### Gene annotation

A total of 662.85 M read pairs were obtained for expression evidence, with individual samples ranging from 50.41 M to 72.67 M read pairs. The evidence-based annotation derived from RNA-seq datasets was merged with the deep learning prediction to produce a unified annotation dataset. The final gene features were standardized using AGAT 1.3.3, and a total of 97,915 protein-coding genes were identified in the final assembly, with an average protein length of 385.9 amino acids (Table S2). A slight asymmetry in gene counts was observed across the subgenomes: SG2 had the highest count (33,028), followed by SG1 (32,888), and SG3 had the lowest (31,999) (Table S2).

### Completeness of the assembly

The completeness of the assembly was assessed using BUSCO scores for the genome sequences against the Solanales odb10 database (Table S3). The BUSCO score for overall assembly was 99.19%, indicating high completeness. This completeness is primarily driven by the duplicated fraction of genes (97.8%), reflecting the expected retention of core orthologs across the three subgenomes in the hexaploid species. The individual subgenomes (SG1, SG2, and SG3) each showed high levels of complete and single-copy BUSCOs (~ 95%) and low levels of complete and duplicated BUSCOs (~ 2.5%), confirming the successful separation of gene complements into the three near-complete subgenomes. The annotated protein set also showed high completeness (98.9%) for the overall assembly, validating the quality of the gene predictions (Table S3).

### Transposable element density, sequencing coverage, and heterozygosity

**Fig. 2:**
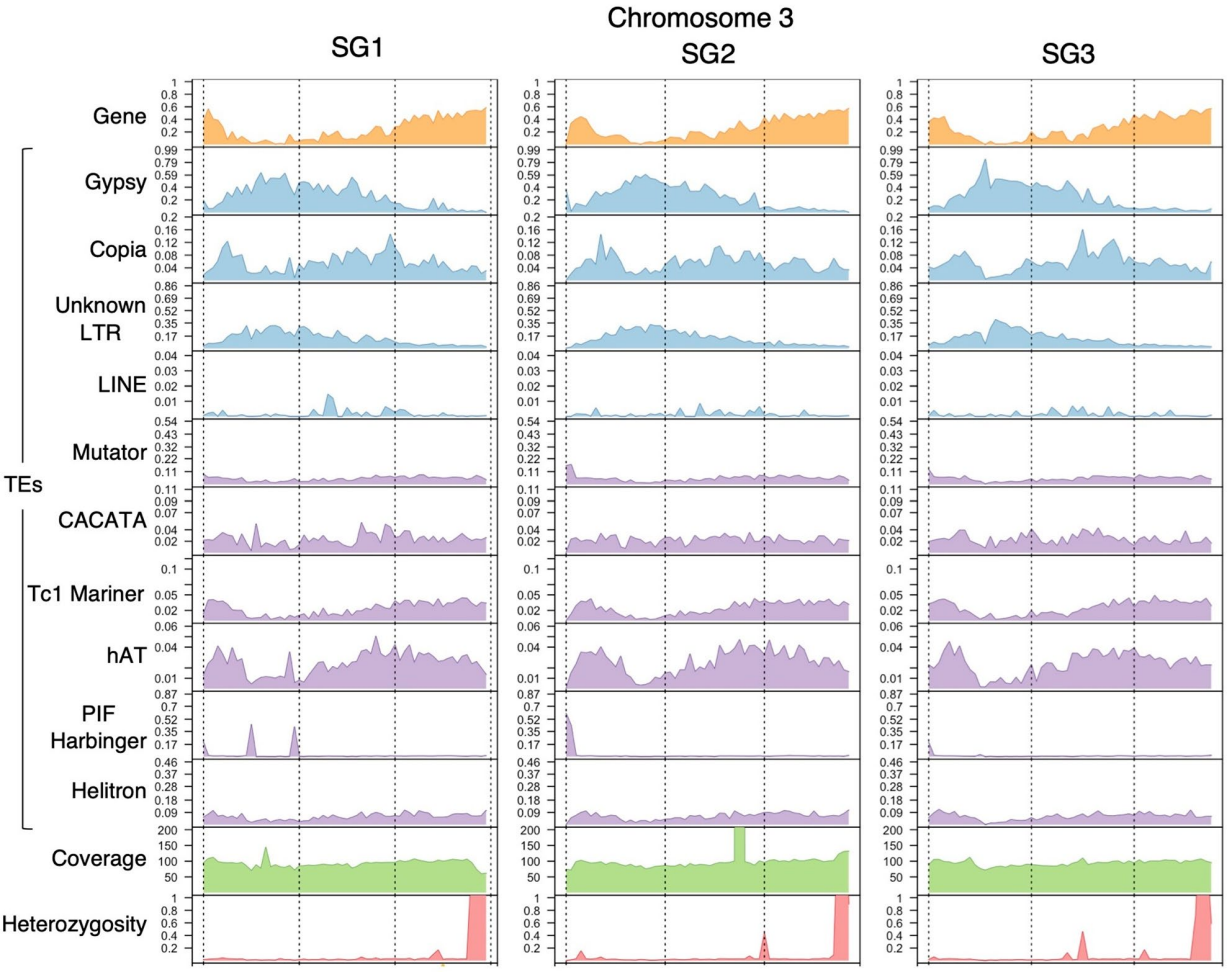
Distributions of genes, various transposable elements (TEs), HiFi-read coverages, and heterozygous portions (heterozygous SNPs/kb) on the *S. demissum* chromosome 3 across the three different subgenomes (SGs)

Transposable elements (TEs) in *S. demissum* were annotated, and genome-wide TE densities were summarized for each subgenome (Table S4). A total of 68.5%, 68.1%, and 67.0% of the SG1, SG2, and SG3, respectively, were occupied by TEs. Gypsy elements were the most abundant TE family across all three *S. demissum* subgenomes (26.0–28.3%). The relative contributions of other major TE families, including Copia and DNA transposons, were also highly similar among subgenomes, and no pronounced subgenome-specific enrichment or depletion of individual TE families was detected. Chromosome-scale visualization of gene density, TE density, sequencing coverage, and heterozygosity revealed conserved structural features among the three subgenomes (Fig. S1). As shown for chromosome 3 as a representative example (Fig. 2), genes were preferentially distributed along chromosome arms, whereas LTR retrotransposons such as Gypsy and Copia (shown in blue) were enriched in gene-poor, pericentromeric regions. In contrast, DNA transposons (shown in purple) were more frequent in gene-rich subtelomeric regions.

Genome-wide coverage of PacBio HiFi reads was largely uniform across chromosomes and subgenomes, indicating that the assemblies do not suffer from major large-scale biases or structural artifacts.

*Solanum demissum* is highly self-fertile, and accordingly, genome-wide heterozygosity was generally very low, with heterozygous alleles rarely detected across most regions of the genome (Fig. S1). However, notable exceptions were observed. Specifically, striking increases in heterozygosity were detected in the distal regions of chromosomes 3 and 4 across all three subgenomes (Fig. 2, Fig. S1). Subgenome-specific increases were also detected in chromosome 1 of SG1, chromosome 5 of SG2, chromosome 7 of SG1 and SG2, and chromosome 9 of SG2 (Fig. S1).

### Subgenome expression bias

**Fig. 3:**
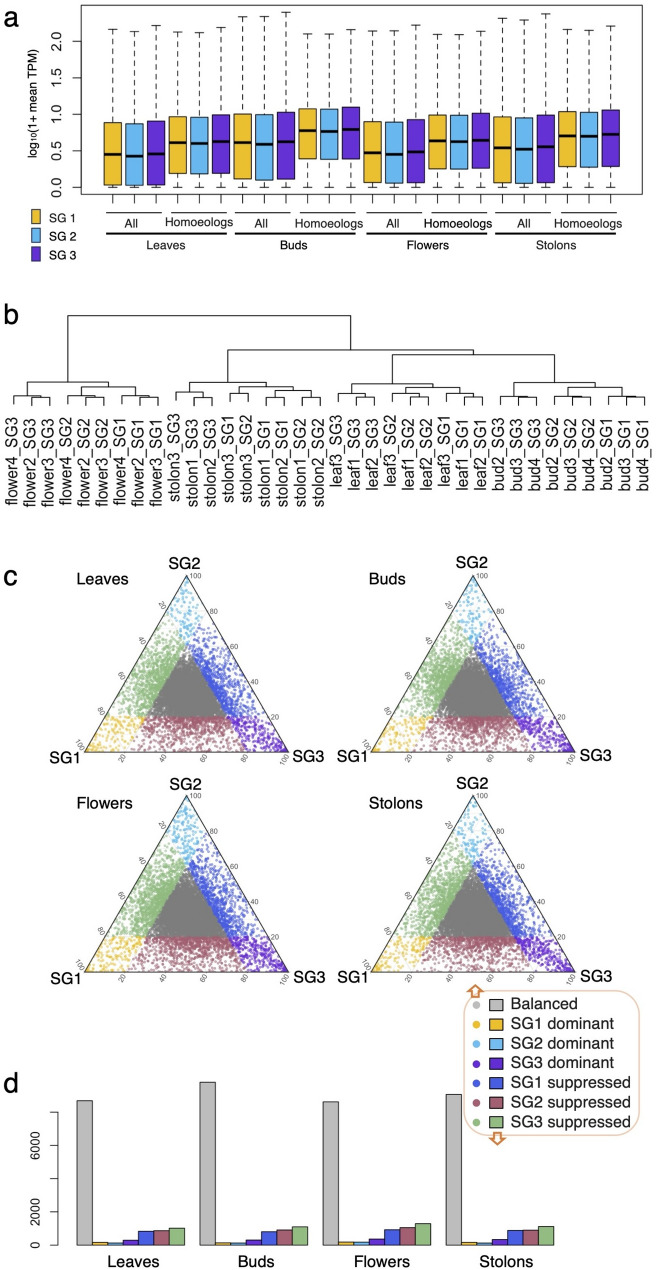
Expression balances among *S. demissum* subgenomes. **a** Boxplots indicate the means and ranges of transcripts per million (TPM) values of three biological replicates for all chromosomal genes (All) and single-copy orthologous gene pairs conserved among the three subgenomes (Homoeologs) in leaves, floral buds, flowers, and stolons, separately plotted for the subgenomes (SGs). **b** A dendrogram based on TPM values of homoeologous genes with the Ward.D2 method. **c** Expression balances of homoeologous genes. **d** The number of homoeologous genes in each category

To evaluate whether the three subgenomes of hexaploid *S. demissum* exhibit transcriptional bias, we first compared expression levels of genes encoded on each subgenome, along with the 15,737 single-copy orthologous gene pairs conserved among the three subgenomes, referred to as homoeologs hereafter, across four tissues (leaf, bud, flower, and stolon) (Table S5). Overall, the distributions of the number of transcripts per million (TPM) values were broadly similar among SG1, SG2, and SG3 for “All genes,” with no subgenome showing obviously higher or lower expression (Fig. 3a). Thus, the three subgenomes are transcriptionally active. For the homoeologs, TPM values were slightly higher than those of all genes across all tissues, consistent with these genes representing a conserved, relatively well-expressed gene set. Even within this subset, differences among SG1, SG2, and SG3 were minimal, suggesting the absence of any strong genome-wide dominance or suppression at the subgenome level.

We next focused on the 15,737 single-copy orthologs shared among all three subgenomes to examine the expression balance of homoeologous triplets in greater detail (Fig. 3b–d). Hierarchical clustering, based on TPM-normalized expression profiles of these triplets, showed highly reproducible grouping of biological replicates (Fig. 3b). Within each tissue cluster, SG1 and SG2 consistently grouped more closely together, whereas SG3 tended to form a slightly more distant branch. Although this separation was subtle, it was stable across tissues. Ternary plots of relative expression among SG1, SG2, and SG3 (Fig. 3c) revealed that most homoeologous triplets were located near the center of the triangle in all tissues, indicating that the three homoeologs generally contributed similar proportions to the total expression of each triplet. In these plots, points near a vertex represent triplets in which one subgenome contributed disproportionately to total expression, whereas points near an edge indicate reduced contribution from one subgenome. Thus, the central enrichment observed across tissues indicates that strongly biased expression was uncommon and that expression was broadly balanced among the three subgenomes. Quantitative classification corroborated this pattern: across tissues, 8,600–9,800 triplets fell into the “Balanced” category, representing the largest category across all tissues (Table S6; Fig. 3d). Here, “Balanced” refers to triplets that did not meet the criteria for subgenome-dominant or subgenome-suppressed expression, indicating the absence of strong expression bias toward or against any one subgenome. Subgenome-dominant or -suppressed classes (SG1-, SG2-, or SG3-dominant/suppressed) each contained fewer than 1,300 triplets. The number of SG3-dominant triplets (297–366) was larger than that of SG1 and SG2 in all tissues (126–185), while the number of SG3-suppressed triplets (1019–1289) was also larger than that of SG1 and SG2 in all tissues (806–1050). Therefore, these comparisons indicate that SG3 has slightly different features in expression profiles than SG1 and SG2.

### Phylogenetic placement of the *S. demissum* subgenomes

To explore the species relationships of *S. demissum*, we conducted a phylogenetic analysis based on gene sequence similarity using 50 genomes and subgenomes with sufficient sequence coverage (Table 1). OrthoFinder classified a total of 1,738,035 genes into 38,509 orthogroups, of which 4,038 were present in all the genomes. Among these common orthogroups, 956 were single-copy orthogroups (Table S7). A maximum-likelihood phylogeny using IQ-TREE 2 was constructed from these 956 single-copy orthogroups.

**Fig. 4:**
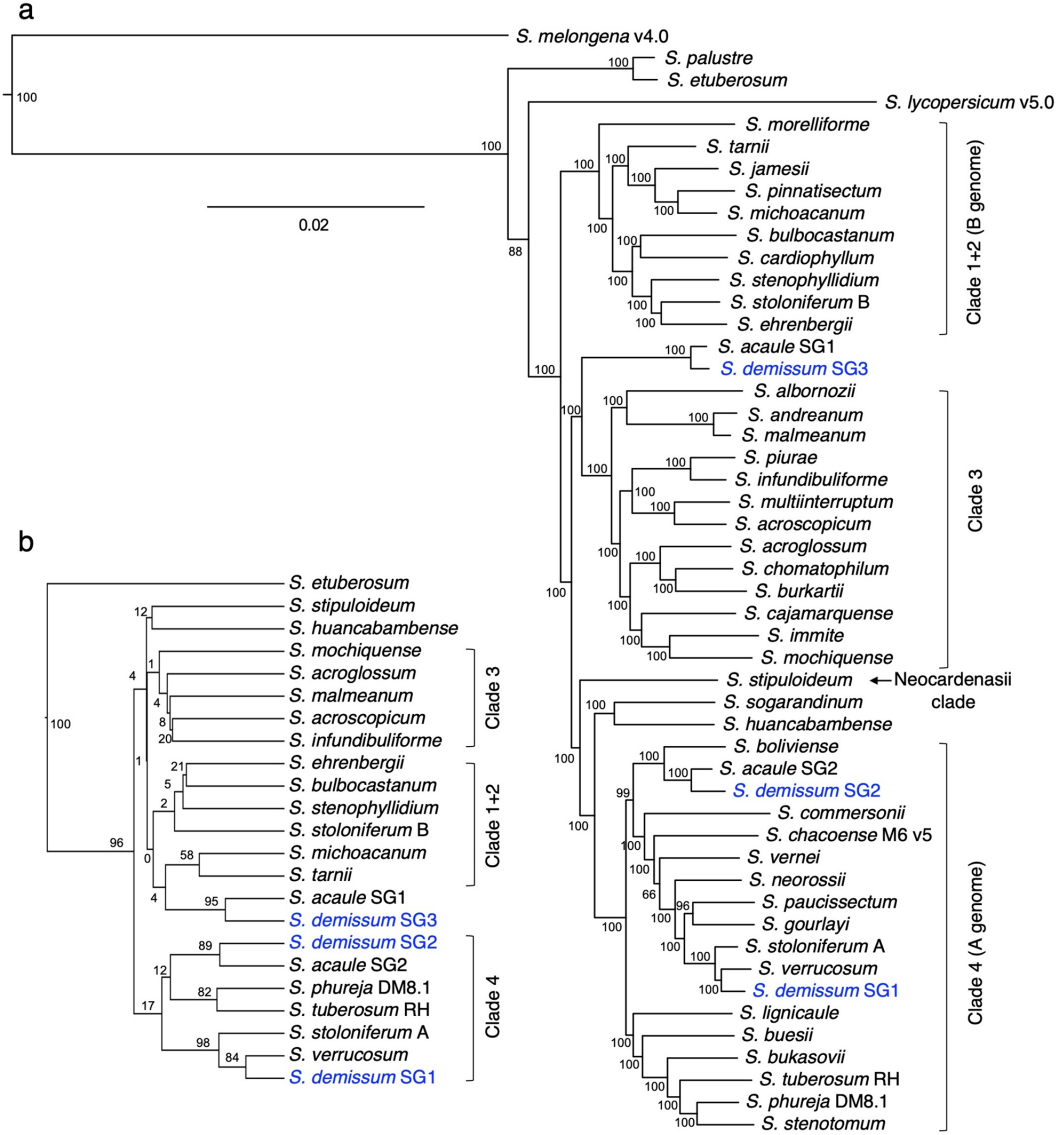
Species relationships based on gene sequence similarity (**a**) and genome structure (**b**). **a** Phylogenetic tree generated using IQ-TREE 2, which was constructed using single-copy gene sequence similarity in 4,038 orthogroups commonly present in all 50 genomes. A bootstrap value is given at each node. **b** Dendrogram was constructed using the UPGMA method, based on pairwise ratios of syntenic regions among 23 chromosome-scale assemblies, showing genome structure similarity

The resulting tree clearly identified three clades among tuber-bearing *Solanum* species (Fig. 4a). The first group comprised Mexican diploid species with the B genome, corresponding to Clade 1+2. The second group comprised *S. piurae* and some other South American species, corresponding to Clade 3. The third group comprised South American A-genome species, including cultivated species and corresponding to Clade 4. *Solanum stipuloideum* Rusby (formerly, *S. circaeifolium* Bitter; [118] clustered as a sister to Clade 4 and to a group containing *S. sogarandinum* Ochoa and *S. huancabambense* Ochoa, supporting the findings by Zhang et al. [148] and the recognition of the’neocardenasii’ clade, which includes *S. stipuloideum* and *S. neocardenasii* Hawkes & Hjert. [125]. Bootstrap support was high for most internal nodes, indicating that the inferred topology is robust.

In the inferred phylogeny, the three *S. demissum* subgenomes were placed distinctly but were closely clustered with other species or genomes of interest. SG1 was closely clustered with the Mexican A-genome diploid species *S. verrucosum* within Clade 4. The *S. verrucosum* + *S. demissum* SG1 clade was clustered with the A genome of *S. stoloniferum* (depicted simply as *S. stoloniferum* A in Fig. 4). SG2 was also placed within Clade 4, but in a distinct clade from SG1 sister to *S. acaule* SG2 (Fig. 4a). This *S. acaule* SG2 + *S. demissum* SG2 clade was clustered with *S. boliviense* (= *S. megistacrolobum*). In contrast, SG3 was most closely clustered with SG1 of *S. acaule* as a lineage sister to Clade 3, forming a distinct branch that did not nest within Clade 3. Thus, *S. demissum* likely comprises genomes from *S. acaule* and *S. verrucosum*.

### Genomic divergence among *S. demissum*, *S. acaule*, and *S. verrucosum*

**Fig. 5:**
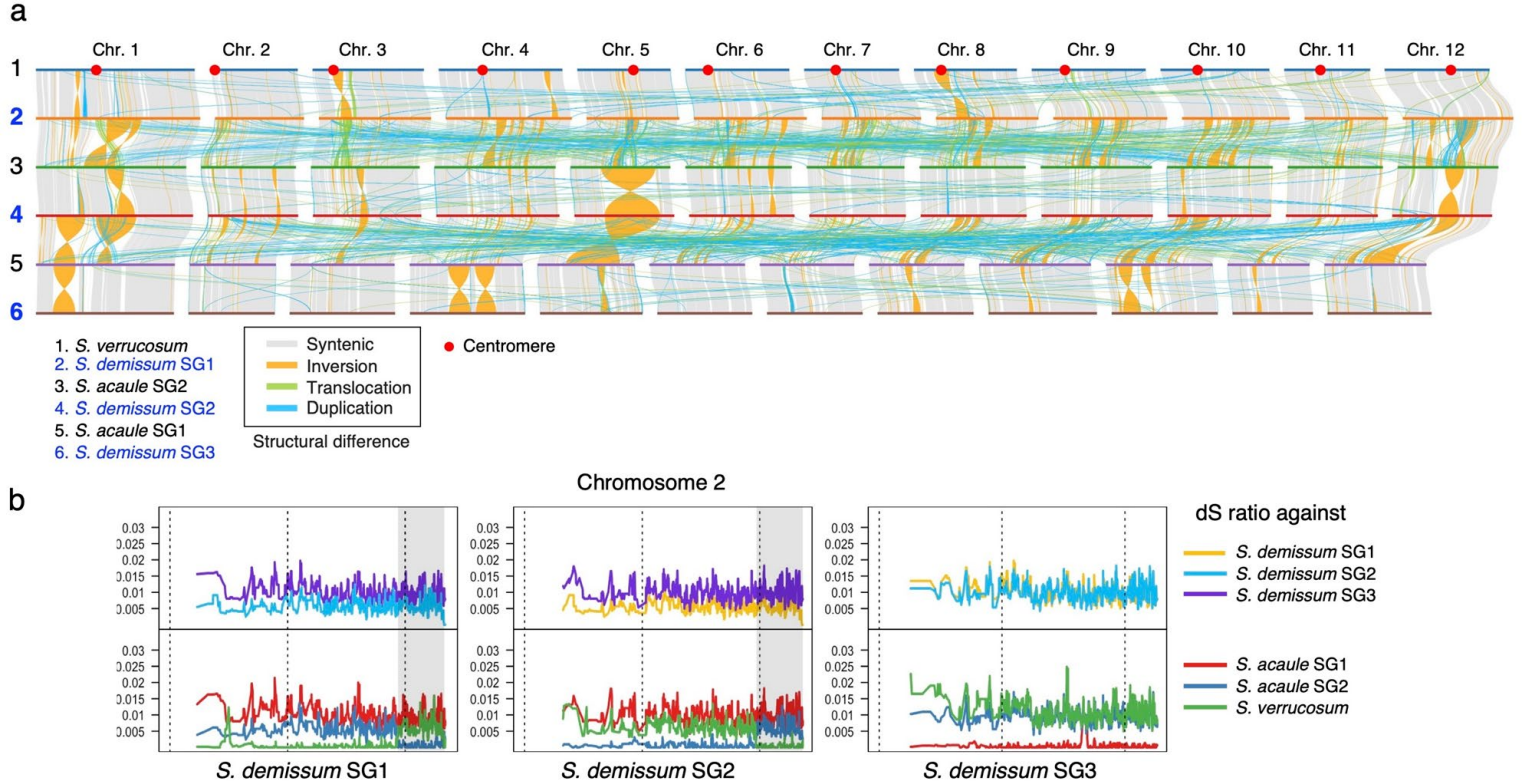
Structural differences among *S. acaule*, *S. demissum*, and *S. verrucosum* genomes. **a** Structural and length variation generated by SyRI and visualized by Plotsr. **b** dS rates plotted along chromosome 2 among the *S. demissum* subgenomes (SGs, upper) and between each *S. demissum* subgenome and the subgenomes of *S. acaule* or *S. verrucosum* (lower). dS values were calculated from single-copy orthologs using a sliding-window approach of 10 consecutive genes ordered by genomic position. Because sliding-window calculation requires a minimum of 10 genes, the plots do not start at the left chromosomal end (0 Mb); the first data point corresponds to the position of the fifth gene, and gene-poor regions near the chromosome end are not represented. Shaded regions indicate chromosomal segments with reduced dS values, consistent with post-origin recombination or homogenization between homoeologous chromosome 2 of SG1 and SG2

To investigate genome structural relationships among *S. demissum*, *S. verrucosum*, and *S. acaule*, we performed a whole-chromosome synteny analysis using SyRI v1.6.3 (Fig. 5a). Large syntenic blocks were broadly conserved between *S. verrucosum* and *S. demissum* SG1, including partial conservation of centromeric regions. Although pericentromeric inversions were observed on chromosomes 3 and 8, the underlying sequences were largely retained. Although there are large pericentromeric inversions in chromosomes 1, 5, and 12 between *S. demissum* SG2 and *S. acaule* SG2, as well as in chromosomes 1, 4, and 10 between *S. demissum* SG3 and *S. acaule* SG1, extensively conserved synteny was observed. In contrast, comparisons between *S. demissum* SG1 and *S. acaule* SG2, and between *S. demissum* SG2 and *S. acaule* SG1, showed numerous duplicated and translocation connections (visualized as dense blue and green links) and, in addition, small and large inversions.

To further assess sequence-level divergence, we estimated pairwise synonymous substitution (dS) rates among *S. demissum*, *S. verrucosum*, and *S. acaule* using single-copy orthologs and visualized chromosome-scale patterns (Fig. S2; Fig. 5b). Across most chromosomes, dS distributions were consistent with the phylogenetic relationships inferred by OrthoFinder. For example, chromosome-wide dS rates among *S. demissum* subgenomes were generally higher between SG3 and SG1 or SG2 than between SG1 and SG2, indicating greater sequence divergence of SG3 relative to SG1 and SG2. In addition, pairwise comparisons between *S. demissum* SG1 and *S. verrucosum* or *S. acaule* genomes revealed clear differences in divergence levels: dS rates between SG1 and *S. verrucosum* were consistently low, whereas comparisons with *S. acaule* SG2 showed moderately higher dS rates, and those with *S. acaule* SG1 were higher. Likewise, dS rates between SG2 and *S. acaule* SG2, and between SG3 and *S. acaule* SG1, were consistently lower than those for the other comparisons.

However, several notable local deviations were detected. *Solanum demissum* SG1 exhibited exceptionally low dS rates against *S. acaule* SG2 and high dS rates against *S. verrucosum* in the right terminal region of chromosome 2, whereas *S. demissum* SG2 exhibited the opposite pattern (Fig. 5b). This is likely caused by recombination between homoeologous chromosome 2 of SG1 and SG2, which occurred after *S. demissum* originated. On the right terminal region of chromosome 3, *S. demissum* SG1 against SG2 and SG2 against SG1 exhibited exceptionally low dS rates, indicating an unusually high sequence similarity between these regions (Fig. S2). In the same genomic interval, dS rates between SG1 and *S. acaule* SG2 were also markedly reduced. In addition, partial regions on chromosomes 1, 4, 6, 9, 10, and 11 of all three subgenomes displayed markedly elevated dS rates when compared against *S. verrucosum* (Fig. S2). These regions likely represent segments where the expected *S. verrucosum*-derived signal has been disrupted, indicating accelerated sequence divergence in *S. verrucosum* after *S. demissum* originated. We note that local gene-model disruption or partial CDS-prediction errors may also contribute to inflated dS estimates in some regions.

### Comparison of the genome structures of tuber-bearing *Solanum* species

Genome structure similarity was investigated using pairwise ratios of syntenic regions obtained by SyRI. Although 30 chromosome-scale assemblies, out of 50 genomes used to evaluate gene sequence similarity, were loaded into SyRI, only 23 could be analyzed due to overload or other technical issues (Table S8). An average syntenic region with *S. etuberosum* was 33%, while those of the others ranged from 40s to 60s% (Table S8). The syntenic region between *S. tuberosum* RH and *S. phureja* DM8.1 was 72%. In contrast, syntenic regions between *S. demissum* SG1 and *S. verrucisum* (85%), between *S. demissum* SG2 and *S. acaule* SG2 (73%), and between *S. demissum* SG3 and *S. acaule* SG1 (76%) were the highest among all the others. Using the pairwise ratios, a dendrogram was constructed by the UPGMA method (Fig. 4b). Three large clusters were identified, corresponding to Clades 1+2, 3, and 4, similar to the results of gene sequence similarity (Fig. 4a). However, bootstrap support for higher-order topology was extremely low. The genome structures of *S. demissum* SG1, SG2, and SG3 were distinct from each other and most closely clustered with those of *S. verrucosum*, *S. acaule* SG2, and *S. acaule* SG1, respectively. The clusters of *S. demissum* SG2 and *S. acaule* SG2, and of *S. demissum* SG1 and *S. verrucosum*, were positioned within Clade 4, consistent with the gene-similarity-based phylogeny. However, the cluster comprising *S. demissum* SG3 and *S. acaule* SG1, which was most closely related to Clade 3 in the gene-similarity-based phylogeny, was grouped with Clade 1+2, although bootstrap support was extremely low. *Solanum stipuloideum* and *S. huancabambense* were distinct from the others and sister to the cluster of Clade 1+2 and Clade 3 with extremely low bootstrap support.

### Transposable element (TE) landscape in *Solanum demissum*

**Fig. 6:**
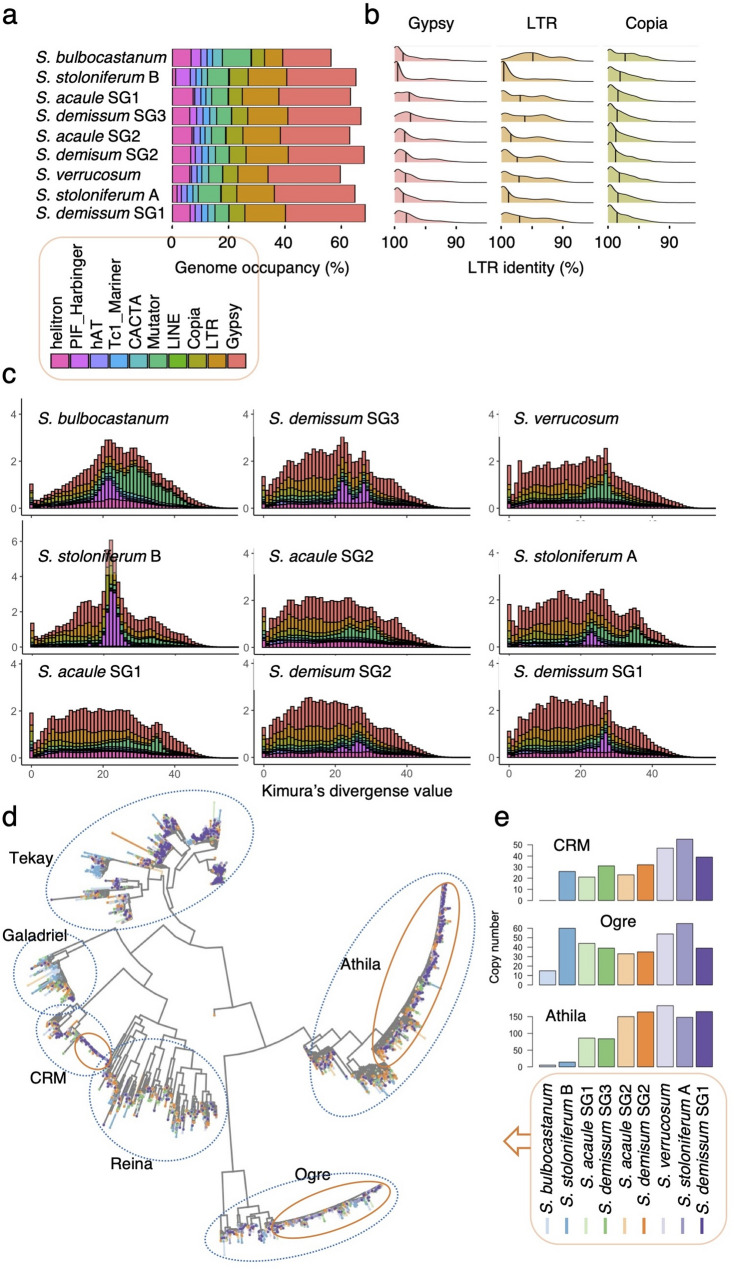
Dynamics of transposable elements (TEs) compared among genomes and subgenomes (SGs) of *S. bulbocastanum*, *S. stoloniferum*, *S. acaule*, *S. demissum*, and *S. verrucosum*. **a** Genome occupancy of the TEs. **b** Density plots of LTR identities in prominent LTR-type retrotransposon families, Gypsy, LTR, and Copia. Vertical lines indicate median values. c Kimura’s divergence values against consensus TE sequences. d Phylogenetic tree of Gypsy retrotransposons inferred by VeryFastTree from 6,894 RH-RT-INT concatenated sequences. The terminal nodes are colored based on the genomes from which their sequences are derived. Three rapidly diversifying clades were identified within the Gypsy families, Athila, Ogre, and CRM, as indicated by orange circles. e Copy numbers of TEs in each group of the rapidly diversifying clades

To characterize the TE landscape of *S. demissum*, we compared its TE composition with those of tetraploid species, *S. acaule* and *S. stoloniferum*, and diploid species, *S. bulbocastanum* (B genome) and *S. verrucosum* (A genome). The subgenomes of polyploid species tended to show somewhat higher total TE occupancy (63.0–68.5%) than diploid species (56.4 and 59.7%) (Fig. 6a; Table S9).

Analyses of the LTR-type retrotransposon families, Gypsy, Copia, and unclassified LTR retrotransposons showed that, unlike the B genome of *S. stoloniferum*, which is characterized by an excess of very young Gypsy and LTR elements, *S. demissum* and *S. acaule* subgenomes did not exhibit pronounced enrichment of high-identity TE elements (Fig. 6b). Lower Kimura divergence values indicate young TEs, or recently inserted TEs, whereas higher values indicate old TEs (Fig. 6c). The B genome of *S. stoloniferum* has experienced rapid diversification or a burst of PIF Harbinger-type DNA transposons, with a sharp peak at approximately Kimura divergence value of 22. All three subgenomes of *S. demissum* exhibit two peaks of PIF Harbinger-type DNA transposons, though not as high as the B genome of *S. stoloniferum*, at approximately Kimura divergence values of 22 and 28, suggesting that there were TE bursts twice in their histories (Fig. 6c).

The phylogenetic tree was inferred from 6,894 predicted protein sequences representing the RNase H (RH), reverse transcriptase (RT), and integrase (INT) domains encoded by Gypsy copies. Gypsy subfamily assignments were obtained using TEsorter, and the sequences were grouped into Athila, Ogre, Reina, CRM, Galadriel, and Tekay (Fig. 6d). Three highly diverse yet little differentiated Gypsy sequence groups with high copy numbers and short branch lengths were found in the Athila, Ogre, and CRM families (Fig. 6d). The copy numbers within these clades (Fig. 6e, Table S10) indicate that Gypsy copies from *S. bulbocastanum* (B genome) were rarely found in these rapidly diversifying clades. However, the B genome of *S. stoloniferum* showed copy numbers of CRM and Ogre families roughly comparable to those of the other genomes, but, like *S. bulbocastanum*, few copy numbers of the Athila family differed from the others. SG1, SG2, and SG3 of *S. demissum* showed approximately the same numbers as those of *S. verrucosum*, *S. acaule* SG2, and *S. acaule* SG1, respectively, for Gypsy copies of CRM, Ogre, and Athila families.

### Resistance gene repertoire and chromosomal distribution in *S. demissum*

**Fig. 7:**
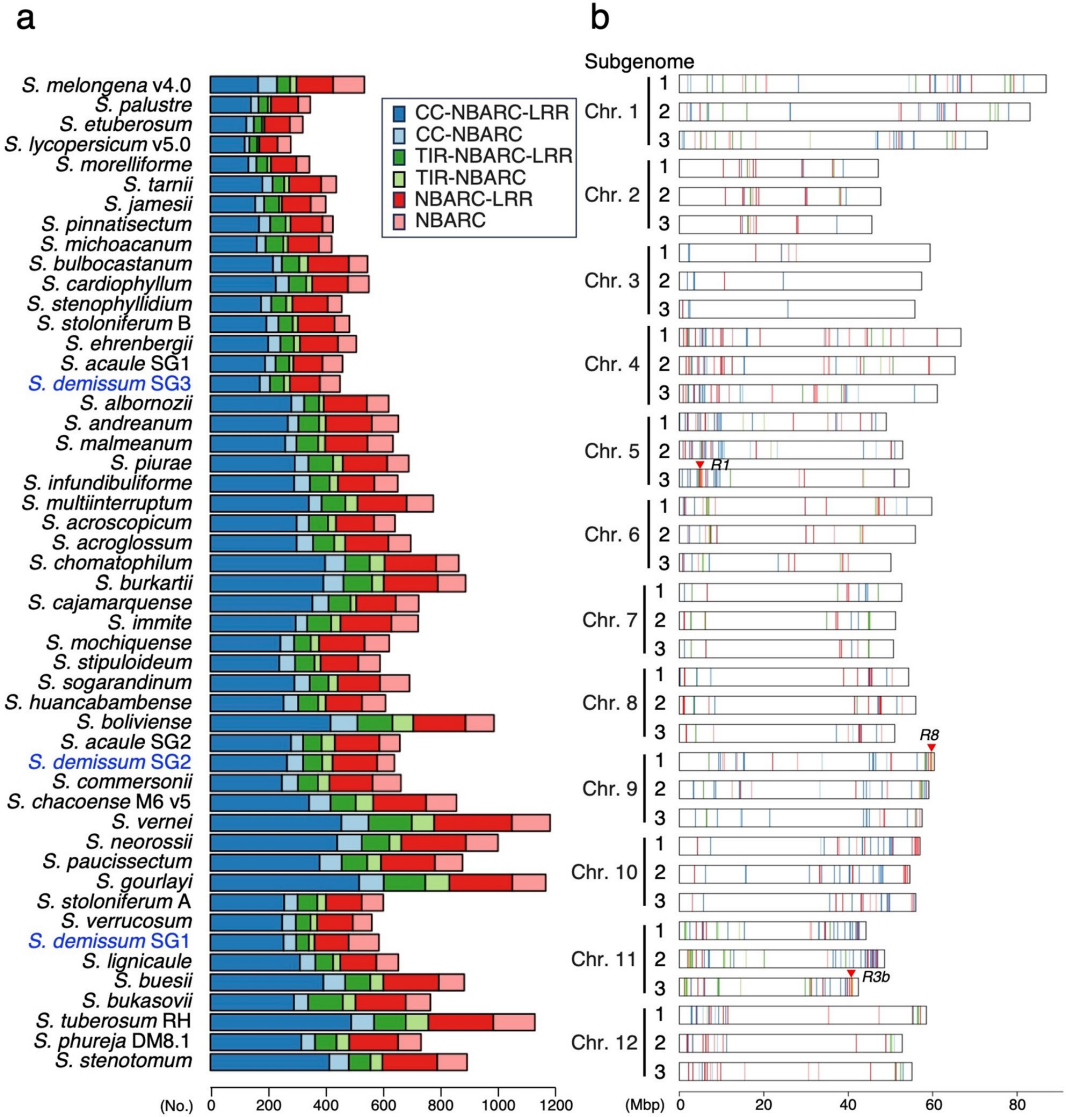
Resistance (*R*) genes. **a** Comparison of nucleotide-binding and leucine-rich repeat (NLR) gene copy numbers across *Solanum* species and subgenomes (SGs). Bars indicate the total number of predicted NLR genes in each genome or subgenome. **b** Chromosomal distribution of NLR genes in *S. demissum*. Positions of previously characterized late blight resistance genes (*R1*, *R3b*, and *R8*) identified in this clone are indicated as red triangles

Several types of putative resistance (*R*) genes were identified among the 50 *Solanum* genomes and subgenomes (Fig. 7a). Total number of predicted *R* genes ranged from 281 (*S. lycopersicum* v5.0) to 1187 (*S. vernei* Bitter & Wittm.) (Table S11) and was significantly lower for diploid species in Clade 1+2 (mean of 459.9) than those of the species in Clade 3 and Clade 4 (means of 709.9 and 810.6, respectively) (*t*-test, *P* < 0.0001). The most common type was CC-NBARC-LRR, followed by NBARC-LRR, which were broadly similar across the 50 genomes and subgenomes. SG1, SG2, and SG3 of *S. demissum* contained 589, 643, and 453 *R* genes, respectively, which were approximately comparable to those of *S. verrucosum* (564), *S. acaule* SG2 (662), and *S. acaule* SG1 (462), respectively (Table S11). Thus, the three subgenomes have largely retained their inherited *R*-gene complements, and no subgenome-specific expansion or contraction occurred following polyploidization. Within *S. demissum*, *R* genes were unevenly distributed along chromosomes, forming distinct clusters (Fig. 7b).

The gene sequences of the five *S. demissum*-derived late blight resistance genes *R1*, *R2*, *R3a*, *R3b*, and *R8* were searched using blastp [12] against the *S. demissum* assembly. *R1*, *R3b*, and *R8* were found with > 99% homology, located on chromosome 5 of SG3, chromosome 11 of SG3, and chromosome 9 of SG1, respectively (Fig. 7b). *R2* and *R3a* were not found in the present assembly with high homology.

## Discussion

### Subgenomes of *S. demissum*

We sequenced three subgenomes (SG1, SG2, and SG3) of the hexaploid species *S. demissum* using PacBio long-read sequencing and Hi-C scaffolding technologies. These subgenomes range from 653 to 697 Mb in size and contain various TEs, along with chromosomal distributions of TEs and gene density, similar to previous reports in *Solanum* [4, 10, 30, 47, 48, 50, 144]. Higher TE contents are featured in subgenomes of polyploid species than in diploid species [48, 88]. Despite the predominantly homozygous background, common heterozygosity peaks across all three subgenomes and subgenome-specific heterozygosity peaks were found, suggesting that these regions may be subject to evolutionary constraints that favor the maintenance of allelic diversity, such as balancing selection, rather than being explained solely by recombination between homoeologous chromosomes or technical artifacts.

### Genomic or species origin of *S. demissum*

The gene sequence-based phylogenetic tree clearly identified three clades among tuber-bearing *Solanum* species, consistent with the well-established three-clade structure of the Petota clade [3, 53, 76, 108, 122, 131, 148]. High similarity in both gene sequences and genome structure was observed between *S. demissum* SG1 and *S. verrucosum*, between *S. demissum* SG2 and *S. acaule* SG2, and between *S. demissum* SG3 and *S. acaule* SG1 (Fig. 4). Thus, we hypothesize that *S. demissum* originated from a cross between *S. verrucosum* as the female parent and *S. acaule* as the male parent. *Solanum verrucosum* is cross-compatible with most South American species as the female parent [24] and with some Mexican diploid species [42, 57]. *Solanum acaule* is also known as a bridging species between A- and B-genome species [21, 41]. The endosperm balance number (EBN) hypothesis [60] states that EBN is a species-specific genetic factor, independent of ploidy, that controls endosperm development in interspecific crosses. *Solanum verrucosum* and *S. acaule* have a balanced EBN value of 2 [36]. Therefore, the cross between *S. verrucosum* and *S. acaule* could be successful, yet results in sterile triploid hybrids. To overcome sterility, unreduced gametes or 2*n* gametes likely contributed to polyploidization because high rates of 2*n* pollen-producing plants were reported in *S. acaule*, *S. verrucosum*, and *S. demissum* [135]. Additivity of EBN [23] yields an EBN value of 4, which matches that of *S. demissum* [36].

A close relationship between *S. demissum* and *S. acaule* has long been recognized. However, *S. verrucosum* and *S. demissum* are distributed in Mexico, while *S. acaule* is distributed from Ecuador to northwest Argentina [38, 123]. There is no geographical overlap in their current distribution ranges. For this reason, no one considered *S. acaule* to be involved in the origin of *S. demissum*, except Spooner et al. [120], who proposed three hypotheses: convergence, a common ancestor, and a progenitor-derivative relationship to explain the similarities between *S. demissum* and *S. albicans*. The common ancestor hypothesis suggests that *S. acaule* is the common ancestor of *S. demissum* and *S. albicans*. The potential for long-distance dispersal of *S. acaule* is suggested, since the distance between the southern distribution of *S. demissum* in Guatemala and the northern distribution of *S. acaule* in Ecuador is approximately 1300 km [120], which is much shorter for the Mexican diploid species *S. morelliforme* Bitt. & Muench, which was discovered 4000 km apart in Bolivia [115]. Also, the wide distribution of *S. acaule*, from Ecuador to northern Argentina, demonstrates its dispersal ability [120] or suggests a previous expansion of its distribution area further north during glacial periods because *S. acaule* is adapted to very high altitudes over 3700 m [38]. An A-genome species or *S. verrucosum* migrated from South America to Mexico [38] and generated an allotetraploid species *S. stoloniferum* with a Mexican B-genome species [48]. Nevertheless, the A genome of *S. verrucosum* was most closely clustered to *S. demissum* SG1 rather than to *S. stoloniferum* A or other A-genome species (Fig. 4), suggesting that the hybridization between *S. verrucosum* and *S. acaule* occurred recently, not on the way to the migration of *S. verrucosum* to Mexico.

### Association between meiotic chromosome pairing and genomic homology

The distinctiveness among the three subgenomes of *S. demissum* obviously results in regular meiosis with 36 bivalents [79, 85, 130]. SG1 and SG2 of *S. demissum* were placed within Clade 4, whereas SG3 was distinctly clustered either with Clade 3 or Clade 1+2 (Fig. 4). This relationship among the three subgenomes of *S. demissum* is consistent with previously observed chromosome behaviors at meiosis. Relatively high frequencies of bivalents (4.74 to 9.78) and univalents in the triploid haploid clones of *S. demissum* [5, 22, 52, 56, 79] indicate that *S. demissum* has two somewhat similar genomes, namely SG1 and SG2, and a third, rather different one, SG3.

*Solanum acaule* shows regular meiosis with 24 bivalents, indicating that the two genomes, *S. acaule* SG1 and SG2, are distinct [56, 83, 129]. This supports the idea that *S. demissum* SG3 (or *S. acaule* SG1) differs from *S. demissum* SG2 (or *S. acaule* SG2) and SG1. As *S. demissum* SG1 is similar to the A genome of *S. verrucosum*, the A genome should be assigned to *S. demissum* SG1 and SG2, different from the previous interpretation that *S. demissum* SG3 is an A genome, different from SG1 and SG2, as indicated by Matsubayashi’s formula AADDD^d^D^d^ [56, 65, 79, 85, 102]. Matsubayashi [84] obtained a heptaploid hybrid (2*n* = 84) from *S. acaule* × *S. demissum*, likely resulting from fertilization of a 2*n* egg of *S. acaule* with *n* pollen of *S. demissum*. He reported that the mean pairing frequency of the hybrid was 0.18 V + 1.11IV + 11.73III + 18.11II + 7.26I per cell, with 12III + 20II + 8I as the modal configuration. This pairing frequency was interpreted based on his genome formula [84], but can also be understood in light of our current understanding that *S. demissum* SG1 and SG2 are A genomes. Therefore, chromosome pairing behaviors clearly reflect similarities in genome sequence and structure. The genome structures of *S. demissum* SG3 and *S. acaule* SG1 are more similar to those of B-genome species (Fig. 4b). However, gene sequence similarity indicates that these two genomes are sister to Clade 3 species, as previously shown for *S. acaule* SG1 [1, 11]. GISH analysis also indicated that signals with the B-genome probe were absent in *S. acaule* and *S. demissum* [98]. Therefore, *S. demissum* SG3 and *S. acaule* SG1 are clearly not B genomes but are closely related to the genomes in Clade 3. The species in Clade 3 are sometimes represented as P-genome species [53, 85, 98, 108, 122]. However, there have been too few chromosome-pairing observations to assign a genome symbol to Clade 3 species and to *S. demissum* SG3.

### Subgenomic differentiation after hexaploidization

Three subgenomes of *S. demissum* have not undergone subfunctionalization and are active, with no clear expression biases, as most homoeologous genes exhibit balanced expression, consistent with the results in an allotetraploid *S. stoloniferum* [48] and other allopolyploid crop species such as cotton [147], *Brassica* [14, 140], and peanut [142]. Achakkagari et al. [1] also reported a balanced expression pattern between the subgenomes of *S. acaule*. However, subtle expression bias toward more dominant and suppressed expression was observed in SG3, consistent with its phylogenetic position, which is more divergent from SG1 and SG2, reflecting underlying evolutionary history rather than a pronounced expression-level bias. TE compositions and genome occupancy frequencies are also similar across the three subgenomes, but they are more similar to those of the ancestral genomes in *S. acaule* and *S. verrucosum*. Subgenome-specific TE bursts, previously observed in the B genome of the allotetraploid species *S. stoloniferum* [48], were not observed in any subgenomes of *S. demissum*. Although recent TE bursts have been detected in the Gypsy families of *S. demissum* subgenomes, they are inherited from the ancestral genomes in *S. acaule* and *S. verrucosum*. Thus, TE activities in response to genome shock [86] at the origin of *S. demissum* were likely minimal. The synteny and dS analyses demonstrated that, while the overall genome architecture of *S. demissum* largely preserves the structural features of its progenitor genomes, its chromosomes have undergone extensive localized remodeling after polyploid formation (Fig. 5). For example, the dS analysis uncovered recombination that occurred after *S. demissum* originated, between homoeologous chromosomes, resulting in the exchange of terminal segments between chromosome 2 of SG1 and SG2, and in the duplication of terminal segments in chromosome 3 of SG1 and SG2.

### *S. demissum* armed by quantity rather than quality of *R* genes?

Consistent with previous studies [6, 61, 136], the most common type of *R* genes was CC-NBARC-LRR, followed by NBARC-LRR, which were broadly similar across the 50 genomes and subgenomes. These putative *R* genes were unevenly distributed along chromosomes, forming distinct clusters, consistent with the locations of NBARC-LRR genes reported by Jupe et al. [61], which are known as resistance gene hotspots [31]. The Mexican highlands are the center of diversity for *P. infestans* [93], the causal pathogen of late blight, the most devastating disease in potatoes. Thus, Mexican wild species, including *S. demissum*, have coevolved and survived with *P. infestans* to date. Mexican diploid species have fewer *R* genes (mean of 459.9) than Clade 3 and Clade 4 species (mean of 709.9 and 810.6, respectively). *Solanum demissum* has a 3.7 times greater number of *R* genes (1685), equivalent to the sum of *R* genes in the parental genomes in *S. verrucosum* and *S. acaule*. The identified late blight resistance genes in the present *S. demissum* clone are located on chromosomes 5 and 11 of SG3 (*R1* and *R3b*) and chromosome 9 of SG1 (*R8*) in similar locations to those originally reported [54, 59, 71]. Thus, not a specific subgenome supplied late blight resistance genes, but all subgenomes likely did. It is known that any single clone of *S. demissum* has at least eight resistance genes to late blight and protects itself to the level of coexistence with *P. infestans* in natural habitats [105, 106]. Thus, many *R* genes from different subgenomes act in a complementary manner to protect *S. demissum* against *P. infestans*. In turn, during the past decades, strong and broad-spectrum late blight resistance genes have been identified from Mexican diploid species: *RB*/*Rpi-blb1*, *Rpi-blb2*, *Rpi-blb3*, and *Rpi-abpt* from *S. bulbocastanum* [78, 116, 132, 133], and *Rpi1* and *Rpi-blb3* from *S. pinnatisectum* [70, 112]. *R1* to *R11* were identified from *S. demissum*, but none, as single genes, were durable; their resistance was broken. In contrast, resistance genes isolated from *S. bulbocastanum* or *S. pinnatisectum*, although fewer in number, conferred broad-spectrum resistance against multiple races. A similar pattern might be expected for other pathogens, indicating that Mexican diploid species are useful sources of disease resistance in potato breeding.

## Conclusions

We provided a high-quality de novo chromosome-scale assembly of the allohexaploid species *S. demissum*, which is historically the most popular wild species in potato breeding, and revealed that its genome originated from *S. verrucosum* and *S. acaule*. During the origin of *S. demissum*, parental genomes did not undergo significant rearrangement or reorganization and were maintained almost intact, contrasting with the origin of a Mexican alloteraploid species, *S. stoloniferum* [48]. Due to balanced EBN values between *S. demissum* and *S. tuberosum*, the two species can be crossed easily, though the resulting hybrids are pentaploid, and backcrossed to *S. tuberosum* [111]. This study will provide important insights into polyploid evolution in tuber-bearing *Solanum* species and into the development of a strategy for resistance breeding.

## Supplementary Information

Supplementary Material 1: Fig. S1 Distributions of genes, transposable elements, HiFi-read coverages, heterozygous portions (heterozygous SNPs/kb), and subgenome-specific *k*-mers on the *S. demissum* chromosomes. Fig. S2 Chromosome-by-chromosome dS ratios among the *S. demissum* subgenomes (upper) and between each subgenome of *S. demissum* and the *S. acaule* subgenomes or *S. verrucosum* (lower).

Supplementary Material 2: Table S1 Chromosome lengths of *S. demissum*. Table S2 Annotated genes. Table S3 BUSCO statistics. Table S4 Transposable elements. Table S5 Statistics of the RNA-seq analysis. Table S6 The number of homoeologous genes in each category. Table S7 Statistics of the OrthoFinder analysis. Table S8 Pairwise ratios of syntenic regions. Table S9 Comparison of genome occupancy of the TEs. Table S10 The number of copies in Gypsy sequence groups with high copy numbers and short branch lengths. Table S11 *R* gene count.

## Data Availability

The raw sequencing reads and the assembly of *S. demissum* have been deposited into the National Center for Biotechnology Information under BioProject Number PRJNA1405828. All the other data supporting the reported results are included in the article or the Supplementary Information.

## References

[1] Achakkagari S, Camargo-Tavares JC, Praslickova D, Martini C, Bizimungu B, Anglin NL, et al. Better together: subgenomes for allotetraploid potato wild relative *Solanum acaule* Bitt. reveal origins in *Petota* Clade 3 and 4. Plant Genome. 2025;18:e70095. 40836401 10.1002/tpg2.70095 PMC12368328

[2] Altschul SF, Gish W, Miller W, Myers EW, Lipman DJ. Basic local alignment search tool. J Mol Biol. 1990;215:403–10. 2231712 10.1016/S0022-2836(05)80360-2

[3] Ames M, Spooner DM. Phylogeny of *Solanum* series *Piurana* and related species in *Solanum* section *Petota* based on five conserved ortholog sequences. Taxon. 2010;59:1091–101.

[4] Aversano R, Contaldi F, Ercolano MR, Grosso V, Iorizzo M, Tatino F, et al. The *Solanum commersonii* genome sequence provides insights into adaptation to stress conditions and genome evolution of wild potato relatives. Plant Cell. 2015;27:954–68. 25873387 10.1105/tpc.114.135954 PMC4558694

[5] Bains GS, Howard HW. Haploid plants of *Solanum demissum*. Nature. 1950;4227:795. 10.1038/166795a0 14780261

[6] Bakker E, Borm T, Prins P, van der Vossen E, Uenk G, Arens M, et al. A genome-wide genetic map of NB-LRR disease resistance loci in potato. Theor Appl Genet. 2011;123:493–508. 10.1007/s00122-011-1602-z PMC3135832 21590328

[7] Ballvora A, Ercolano MR, Julia W, Meksem K, Bormann CA, Oberhagemann P, et al. The *R1* gene for potato resistance to late blight (*Phytophthora infestans*) belongs to the leucine zipper/NBS/LRR class of plant resistance genes. Plant J. 2002;30:361–71. 12000683 10.1046/j.1365-313x.2001.01292.x

[8] Barchi L, Rabanus-Wallace MT, Prohens J, Toppino L, Padmarasu S, Portis E, et al. Improved genome assembly and pan-genome provide key insights into eggplant domestication and breeding. Plant J. 2021;107:579–96. 33964091 10.1111/tpj.15313 PMC8453987

[9] Bleasby AJ, Ison JC, Rice PM. EMBOSS administrator’s guide: bioinformatics software management. Cambridge: Cambridge University Press; 2011.

[10] Bozan I, Achakkagari SR, Anglin NL, Ellis D, Tai HH, Strömvik MV. Pangenome analyses reveal impact of transposable elements and ploidy on the evolution of potato species. Proc Natl Acad Sci USA. 2023;120:e2211117120. 37487084 10.1073/pnas.2211117120 PMC10401005

[11] Cai D, Rodríguez F, Teng Y, Ané C, Bonierbale M, Mueller LA, et al. Single copy nuclear gene analysis of polyploidy in wild potatoes (*Solanum* section *Petota*). BMC Evol Biol. 2012;12:70. 22624678 10.1186/1471-2148-12-70 PMC3416576

[12] Camacho C, Coulouris G, Avagyan V, Ma N, Papadopoulos J, Bealer K, et al. BLAST+: architecture and applications. BMC Bioinformatics. 2009;10:421. 20003500 10.1186/1471-2105-10-421 PMC2803857

[13] Camadro EL, Masuelli RW, Cortés MC. Haploids of the wild tetraploid potato *Solanum acaule* ssp. *acaule*: generation, meiotic behavior, and electrophoretic pattern for the aspartate aminotransferase system. Genome. 1992;35:431–5.

[14] Chalhoub B, Denoeud F, Liu S, Parkin IA, Tang H, Wang X, et al. Early allopolyploid evolution in the post-Neolithic *Brassica napus* oilseed genome. Science. 2014;345:950–53. 10.1126/science.1253435 25146293

[15] Cheng H, Concepcion GT, Feng X, Zhang H, Li H. Haplotype-resolved *de novo* assembly using phased assembly graphs with hifiasm. Nat Methods. 2021;18:170–5. 33526886 10.1038/s41592-020-01056-5 PMC7961889

[16] Chen S, Zhou Y, Chen Y, Gu J. Fastp: an ultra-fast all-in-one FASTQ preprocessor. Bioinformatics. 2018;34:i884–90. 30423086 10.1093/bioinformatics/bty560 PMC6129281

[17] Correll DS. The potato and its wild relatives. Contr Texas Res Found Bot Stud. 1962;4:1–606.

[18] Danecek P, Bonfield JK, Liddle J, Marshall J, Ohan V, Pollard MO, et al. Twelve years of SAMtools and BCFtools. GigaSci. 2021;10:1–4. 10.1093/gigascience/giab008 PMC7931819 33590861

[19] Debener T, Salamini F, Gebhardt C. Phylogeny of wild and cultivated *Solanum* species based on nuclear restriction fragment length polymorphisms (RFLPs). Theor Appl Genet. 1990;79:360–8. 24226355 10.1007/BF01186080

[20] Dionne LA. Cytoplasmic sterility in derivatives of *Solanum demissum*. Am Potato J. 1961;38:117–20.

[21] Dionne LA. Studies on the use of *Solanum acaule* as a bridge between *Solanum tuberosum* and species in the series *Bulbocastana*, *Cardiophylla* and *Pinnatisecta*. Euphytica. 1963;12:263–9.

[22] Dodds KS. Polyhaploids of *Solanum demissum*. Nature. 1950;4227:795. 10.1038/166795b0 14780262

[23] Ehlenfeldt MK, Hanneman RE Jr. Genetic control of Endosperm Balance Number (EBN): three additive loci in a threshold-like system. Theor Appl Genet. 1988;75:825–32.

[24] Eijlander R, ter Laak W, Hermsen JGTh, Ramanna MS, Jacobsen E. Occurrence of self-compatibility, self-incompatibility and unilateral incompatibility after crossing diploid *S. tuberosum* (SI) with *S. verrucosum* (SC): I. Expression and inheritance of self-compatibility. Euphytica. 2000;115:127–39.

[25] Emms DM, Kelly S. OrthoFinder: phylogenetic orthology inference for comparative genomics. Genome Biol. 2019;20:238. 31727128 10.1186/s13059-019-1832-y PMC6857279

[26] FAOSTAT. 2024. https://www.fao.org/faostat/en/#home. Accessed 13 February 2016.

[27] Faust GG, Hall IM. SAMBLASTER: fast duplicate marking and structural variant read extraction. Bioinformatics. 2014;30:2503–5. 24812344 10.1093/bioinformatics/btu314 PMC4147885

[28] Fry W. *Phytophthora infestans*: the plant (and *R* gene) destroyer. Mol Plant Pathol. 2008;9:385–402. 18705878 10.1111/j.1364-3703.2007.00465.x PMC6640234

[29] Gagnon E, Hilgenhof R, Orejuela A, McDonnell A, Sablok G, Aubriot X, et al. Phylogenomic discordance suggests polytomies along the backbone of the large genus *Solanum*. Am J Bot. 2022;109:580–601. 35170754 10.1002/ajb2.1827 PMC9321964

[30] Gaiero P, Vaio M, Peters SA, Schranz ME, de Jong H, Speranza PR. Comparative analysis of repetitive sequences among species from the potato and the tomato clades. Ann Bot. 2019;123:521–32. 30346473 10.1093/aob/mcy186 PMC6377101

[31] Gebhardt C, Valkonen JPT. Organization of genes controlling disease resistance in the potato genome. Annu Rev Phytopathol. 2001;39:79–102. 11701860 10.1146/annurev.phyto.39.1.79

[32] Goel M, Schneeberger K. Plotsr: visualizing structural similarities and rearrangements between multiple genomes. Bioinformatics. 2022;38:2922–6. 35561173 10.1093/bioinformatics/btac196 PMC9113368

[33] Goel M, Sun H, Jiao W-B, Schneeberger K. SyRI: finding genomic rearrangements and local sequence differences from whole-genome assemblies. Genome Biol. 2019;20:277. 31842948 10.1186/s13059-019-1911-0 PMC6913012

[34] Haas BJ, Kamoun S, Zody MC, Jiang RHY, Handsaker RE, Cano LM, et al. Genome sequence and analysis of the Irish potato famine pathogen *Phytophthora infestans*. Nature. 2009;461:393–8. 19741609 10.1038/nature08358

[35] Hamilton NE, Ferry M. Ggtern: ternary diagrams using Ggplot2. J Stat Softw. 2018;87:1–17.

[36] Hanneman RE Jr. Assignment of endosperm balance numbers to the tuber-bearing *Solanums* and their close non-tuber-bearing relatives. Euphytica. 1994;74:19–25.

[37] Hawkes JG. A revision of the tuber-bearing Solanums, 2nd ed. Scott Plant Breed Stn Rec;1963.

[38] Hawkes JG. The potato: evolution, biodiversity and genetic resources. London: Belhaven Press; 1990.

[39] Hawkes JG, Hjerting JP. The potatoes of Argentina, Brazil, Paraguay, and Uruguay. London: Oxford University Press; 1969.

[40] Hawkes JG, Hjerting JP. The potatoes of Bolivia; their breeding value and evolutionary relationships. New York: Oxford University Press; 1989.

[41] Hermsen JGTh. Crossability, fertility, and cytogenetic studies in *Solanum acaule* × *Solanum bulbocastanum*. Euphytica. 1966;15:149–55.

[42] Hermsen JGTh, Ramanna MS. Barriers to hybridization of *Solanum bulbocastanum* Dun. and *S. verrucosum* Schlechtd. and structural hybridity in their F_1_ plants. Euphytica. 1976;25:1–10.

[43] Hijmans RJ, Spooner DM. Geographic distribution of wild potato species. Am J Bot. 2001;88:2101–12. 21669641

[44] Hosaka K, Spooner DM. RFLP analysis of the wild potato species, *Solanum acaule* Bitter (*Solanum* sect. *Petota*). Theor Appl Genet. 1992;84:851–8. 10.1007/BF00227396 24201486

[45] Hosaka K, Sanetomo R. Effects of *Solanum demissum* chromosomes on crossability in the backcross progeny to *Solanum tuberosum*. Euphytica. 2018;214:113.

[46] Hosaka AJ, Sanetomo R, Hosaka K. A *de novo* genome assembly of *Solanum verrucosum* Schlechtendal, a Mexican diploid species geographically isolated from other diploid A-genome species of potato relatives. G3: Genes|Genomes|Genetics. 2022;12:jkac166. 10.1093/g3journal/jkac166 PMC9339273 35775942

[47] Hosaka AJ, Sanetomo R, Hosaka K. A de novo genome assembly of *Solanum bulbocastanum* Dun., a Mexican diploid species reproductively isolated from the A-genome species, including cultivated potatoes. G3. 2024;14:jkae080. 10.1093/g3journal/jkae080 PMC11152074 38608140

[48] Hosaka AJ, Sanetomo R, Hosaka K. Allotetraploid nature of a wild potato species, *Solanum stoloniferum* Schlechtd. et Bche., as revealed by whole-genome sequencing. Plant J. 2025;121:e17158. 39585203 10.1111/tpj.17158 PMC11703546

[49] Hosaka K, Ogihara Y, Matsubayashi M, Tsunewaki K. Phylogenetic relationship between the tuberous *Solanum* species as revealed by restriction endonuclease analysis of chloroplast DNA. Jpn J Genet. 1984;59:349–69.

[50] Hosmani PS, Flores-Gonzalez M, van de Geest H, Maumus F, Bakker LV, Schijlen E, et al. An improved de novo assembly and annotation of the tomato reference genome using single-molecule sequencing, Hi-C proximity ligation and optical maps. bioRxiv. 2019. 10.1101/767764.

[51] Howard HW, Swaminathan MS. Species differentiation in the section *Tuberarium* of *Solanum* with particular reference to the use of interspecific hybridization in breeding. Euphytica. 1952;1:20–8.

[52] Howard HW, Swaminathan MS. The cytology of haploid plants *Solanum demissum*. Genetica. 1953;26:381–91. 13142313 10.1007/BF01690622

[53] Huang B, Ruess H, Liang Q, Colleoni C, Spooner DM. Analyses of 202 plastid genomes elucidate the phylogeny of *Solanum* section *Petota*. Sci Rep. 2019;9:4454. 30872631 10.1038/s41598-019-40790-5 PMC6418237

[54] Huang S, Vleeshouwers VG, Werij JS, Hutten RC, van Eck HJ, Visser RG, et al. The *R3* resistance to *Phytophthora infestans* in potato is conferred by two closely linked *R* genes with distinct specificities. Mol Plant-Microbe Interact. 2004;17:428–35. 15077675 10.1094/MPMI.2004.17.4.428

[55] Huang SW, van der Vossen EAG, Kuang HH, Vleeshouwers VGAA, Zhang NW, Borm TJA, et al. Comparative genomics enabled the isolation of the *R3a* late blight resistance gene in potato. Plant J. 2005;42:251–61. 15807786 10.1111/j.1365-313X.2005.02365.x

[56] Irikura Y. Cytogenetic studies on the haploid plants of tuber-bearing *Solanum* species. 2. Cytological investigations on haploid plants and interspecific hybrids by utilizing haploidy (*in Japanese*). Res Bull Hokkaido Natl Agric Exp Stn. 1976;115:1–80.

[57] Jansky S, Hamernik A. The introgression of 2*x* 1EBN *Solanum* species into the cultivated potato using *Solanum verrucosum* as a bridge. Genet Resour Crop Evol. 2009;56:1107–15.

[58] Jia KH, Wang ZX, Wang L, Li GY, Zhang W, Wang XL, et al. SubPhaser: a robust allopolyploid subgenome phasing method based on subgenome-specific *k*-mers. New Phytol. 2022;235:801–9. 35460274 10.1111/nph.18173

[59] Jo KR, Arens M, Kim TY, Jongsma MA, Visser RGF, Jacobsen E, et al. Mapping of the *S. demissum* late blight resistance gene *R8* to a new locus on chromosome IX. Theor Appl Genet. 2011;123:1331–40. 21877150 10.1007/s00122-011-1670-0 PMC3214258

[60] Johnston SA, den Nijs TPM, Peloquin SJ, Hanneman RE Jr. The significance of genetic balance to endosperm development in interspecific crosses. Theor Appl Genet. 1980;57:5–9. 24302359 10.1007/BF00276002

[61] Jupe F, Pritchard L, Etherington GJ, MacKenzie K, Cock PJA, Wright F, et al. Identification and localization of the NB-LRR gene family within the potato genome. BMC Genomics. 2012;13:75. 22336098 10.1186/1471-2164-13-75 PMC3297505

[62] Kardolus JP. Morphological variation within series *Acaulia* Juz. (*Solanum* sect. *Petota*). In: Nee M, Symon DE, Lester RN, Jessop JP, editors. Solanaceae IV. Kew: Royal Botanic Gardens;1999. p. 257–74.

[63] Kardolus JP, van Eck HJ, van den Berg RG. The potential of AFLPs in biosystematics: a first application in *Solanum* taxonomy (Solanaceae). Plant Syst Evol. 1998;210:87–103.

[64] Katoh K, Standley DM. MAFFT multiple sequence alignment software version 7: improvements in performance and usability. Mol Biol Evol. 2013;30:772–80. 23329690 10.1093/molbev/mst010 PMC3603318

[65] Kawakami K, Matsubayashi M. Studies on the species differentiation in the section *Tuberarium* of *Solanum*. V. Genomic affinity between *Solanum verrucosum* and *S. demissum*. Sci Rep Hyogo Univ Agric. 1957;3:17–21.

[66] Kim D, Paggi JM, Park C, Bennett C, Salzberg SL. Graph-based genome alignment and genotyping with HISAT2 and HISAT-genotype. Nat Biotech. 2019;37:907–15. 10.1038/s41587-019-0201-4 PMC7605509 31375807

[67] Kimura M. A simple method for estimating evolutionary rates of base substitutions through comparative studies of nucleotide sequences. J Mol Evol. 1980;16:111–20. 7463489 10.1007/BF01731581

[68] Kovaka S, Zimin AV, Pertea GM, Razaghi R, Salzberg SL, Pertea M. Transcriptome assembly from long-read RNA-seq alignments with StringTie2. Genome Biol. 2019;20:278. 31842956 10.1186/s13059-019-1910-1 PMC6912988

[69] Kriventseva EV, Kuznetsov D, Tegenfeldt F, Manni M, Dias R, Simão FA, et al. OrthoDB v10: sampling the diversity of animal, plant, fungal, protist, bacterial and viral genomes for evolutionary and functional annotations of orthologs. Nucleic Acids Res. 2019;47:D807-11. 30395283 10.1093/nar/gky1053 PMC6323947

[70] Kuhl JC, Hanneman RE Jr. Characterization and mapping of *Rpi1*, a late-blight resistance locus from diploid (1EBN) Mexican *Solanum pinnatisectum*. Mol Genet Genomics. 2001;265:977–85. 11523789 10.1007/s004380100490

[71] Leonards-Schippers C, Gieffers W, Salamini F, Gebhardt C. The *R1* gene conferring race-specific resistance to *Phytophthora infestans* in potato is located on potato chromosome V. Mol Gen Genet. 1992;233:278–83. 1351246 10.1007/BF00587589

[72] Li G, Huang S, Guo X, Li Y, Yang Y, Guo Z, et al. Cloning and characterization of *R3b*; members of the *R3* superfamily of late blight resistance genes show sequence and functional divergence. Mol Plant-Microbe Interact. 2011;24:1132–42. 21649512 10.1094/MPMI-11-10-0276

[73] Li H. Minimap2: pairwise alignment for nucleotide sequences. Bioinformatics. 2018;34:3094–100. 29750242 10.1093/bioinformatics/bty191 PMC6137996

[74] Li H, Durbin R. Fast and accurate short read alignment with Burrows-Wheeler transform. Bioinformatics. 2009;25:1754–60. 19451168 10.1093/bioinformatics/btp324 PMC2705234

[75] Li H, Handsaker B, Wysoker A, Fennell T, Ruan J, Homer N, et al. The sequence alignment/map format and SAMtools. Bioinformatics. 2009;25:2078–9. 19505943 10.1093/bioinformatics/btp352 PMC2723002

[76] Li Y, Colleoni C, Zhang J, Liang Q, Hu Y, Ruess H, et al. Genomic analyses yield markers for identifying agronomically important genes in potato. Mol Plant. 2018;11:473–84. 29421339 10.1016/j.molp.2018.01.009

[77] Liao Y, Smyth GK, Shi W. featureCounts: an efficient general purpose program for assigning sequence reads to genomic features. Bioinformatics. 2013;30:923–30. 24227677 10.1093/bioinformatics/btt656

[78] Lokossou AA, Park TH, van Arkel G, Arens M, Ruyter-Spira C, Morales J, et al. Exploiting knowledge of *R/Avr* genes to rapidly clone a new LZ-NBS-LRR family of late blight resistance genes from potato linkage group IV. Mol Plant-Microbe Interact. 2009;22:630–41. 19445588 10.1094/MPMI-22-6-0630

[79] Marks GE. Cytogenetic studies in tuberous *Solanum* species. I. Genomic differentiation in the group Demissa. J Genet. 1955;53:262–9.

[80] Marks GE. Cytogenetic studies in tuberous *Solanum* species. III. Species relationships in some South and Central American species. New Phytol. 1965;64:293–306.

[81] Matsubayashi M. Studies on the species differentiation in the section *Tuberarium* of *Solanum*. III. Behavior of meiotic chromosomes in F_1_ hybrid between *S. longipedicellatum* and *S. schickii*, in relation to its parent species. Sci Rep Hyogo Univ Agric. 1955;2:25–31.

[82] Matsubayashi M. Studies on the species differentiation in *Solanum*, sect. Tuberarium. VIII. Genomic relationships between* S. demissum* and certain diploid* Solanum* species. Seiken Ziho. 1962;13:57–68.

[83] Matsubayashi M. Species differentiation in *Solanum*, sect. *Petota*. XI. Genomic relationships between *S. acaule* and certain diploid *Commersoniana* species. Sci Rep Fac Agric Kobe Univ. 1982;15:23–33.

[84] Matsubayashi M. Species differentiation in *Solanum* sect. *Petota*. XIII. Meiotic behavior of a heptaploid hybrid from *S. acaule* × *S. demissum* and its bearing on the genomic relationship between the parent species. Sci Rep Fac Agric Kobe Univ. 1984;16:1–9.

[85] Matsubayashi M. Phylogenetic relationships in the potato and its related species. In: Tsuchiya T, Gupta PK, editors. Chromosome engineering in plants: genetics, breeding, evolution, Part B. Amsterdam: Elsevier; 1991. p. 93–118.

[86] McClintock B. The significance of responses of the genome to challenge. Science. 1984;226:792–801. 15739260 10.1126/science.15739260

[87] McDonald BA, Linde C. Pathogen population genetics, evolutionary potential, and durable resistance. Annu Rev Phytopathol. 2002;40:349–79. 12147764 10.1146/annurev.phyto.40.120501.101443

[88] Meca E, Díez CM, Gaut BS. Modeling transposable elements dynamics during polyploidization in plants. J Theor Biol. 2024;579:111701. 38128754 10.1016/j.jtbi.2023.111701

[89] Minh BQ, Schmidt HA, Chernomor O, Schrempf D, Woodhams MD, von Haeseler A, et al. IQ-TREE 2: new models and efficient methods for phylogenetic inference in the genomic era. Mol Biol Evol. 2020;37:1530–4. 32011700 10.1093/molbev/msaa015 PMC7182206

[90] Murtagh F, Legendre P. Ward’s hierarchical agglomerative clustering method: which algorithms implement Ward’s criterion? J Classif. 2014;31:274–95.

[91] Nakagawa K, Hosaka K. Species relationships between a wild tetraploid potato species, *Solanum acaule* Bitter, and its related species as revealed by RFLPs of chloroplast and nuclear DNA. Am J Potato Res. 2002;79:85–98.

[92] Neumann P, Novák P, Hoštáková N, Macas J. Systematic survey of plant LTR-retrotransposons elucidates phylogenetic relationships of their polyprotein domains and provides a reference for element classification. Mob DNA. 2019;10:1. 30622655 10.1186/s13100-018-0144-1 PMC6317226

[93] Niederhauser JS. *Phytophthora infestans*: The Mexican connection. In: Lucas JA, Shattock RC, Shaw DS, Cooks LR, editors. *Phytophthora*. England: Cambridge University Press; 1991. p. 25–45.

[94] Ochoa CM. The potatoes of South America: Bolivia. Cambridge: Cambridge University Press; 1990.

[95] Ono S, Sanetomo R, Hosaka K. Genetic transmission of *Solanum demissum* (2*n*=6*x*=72) chromosomes from a pentaploid hybrid of *S. tuberosum* (2*n*=4*x*=48) into the aneuploid BC1 progeny. Euphytica. 2016;207:149–68.

[96] Ou S, Su W, Liao Y, Chougule K, Agda JRA, Hellinga AJ, et al. Benchmarking transposable element annotation methods for creation of a streamlined, comprehensive pipeline. Genome Biol. 2019;20:275. 31843001 10.1186/s13059-019-1905-y PMC6913007

[97] Pedersen BS, Quinlan AR. Mosdepth: quick coverage calculation for genomes and exomes. Bioinformatics. 2018;34:867–8. 29096012 10.1093/bioinformatics/btx699 PMC6030888

[98] Pendinen G, Spooner DM, Jiang J, Gavrilenko T. Genomic in situ hybridization reveals both auto-and allopolyploid origins of different North and Central American hexaploid potato (*Solanum* sect. *Petota*) species. Genome. 2012;55:407–15. 22594521 10.1139/g2012-027

[99] Pertea G, Pertea M. GFF utilities: GffRead and GffCompare. F1000Res. 2020;9:304. 10.12688/f1000research.23297.1 PMC7222033 32489650

[100] Piñeiro C, Abuín JM, Pichel JC. VeryFastTree: speeding up the estimation of phylogenies for large alignments through parallelization and vectorization strategies. Bioinformatics. 2020;36:4658–9. 32573652 10.1093/bioinformatics/btaa582

[101] Plaisted RL, Hoopes RW. The past record and future prospects for the use of exotic potato germplasm. Am Potato J. 1989;66:603–27.

[102] Propach H. Cytogenetische Untersuchungen in der Gattung *Solanum*, Sect. *Tuberarium*. II. Triploide und tetraploide Artbastarde. Z Indukt Abstammungs- Vererbungsl. 1937;73:143–54.

[103] Quinlan AR, Hall IM. BEDTools: a flexible suite of utilities for comparing genomic features. Bioinformatics. 2010;26:841–2. 20110278 10.1093/bioinformatics/btq033 PMC2832824

[104] Ramanna MS, Hermsen JGTh. Structural hybridity in the series *Etuberosa* of the genus *Solanum* and its bearing on crossability. Euphytica. 1981;30:15–31.

[105] Rivera-Peña A. Wild tuber-bearing species of *Solanum* and incidence of *Phytophthora infestans* (Mont.) de Bary on the western slopes of the volcano Nevado de Toluca. 2. Distribution of *Phytophthora infestans*. Potato Res. 1990;33:341–7.

[106] Rivera-Peña A. Wild tuber-bearing species of *Solanum* and incidence of *Phytophthora infestans* (Mont.) de Bary on the western slopes of the volcano Nevado de Toluca. 3. Physiological races of *Phytophthora infestans*. Potato Res. 1990;33:349–55.

[107] Rodewald J, Trognitz B. *Solanum* resistance genes against *Phytophthora infestans* and their corresponding avirulence genes. Mol Plant Pathol. 2013;14:740–57. 23710878 10.1111/mpp.12036 PMC6638693

[108] Rodríguez F, Spooner DM. Nitrate reductase phylogeny of potato (*Solanum* sect. *Petota*) genomes with emphasis on the origins of the polyploid species. Syst Bot. 2009;34:207–19.

[109] Ross H. Potato breeding: problems and perspectives. Berlin and Hamburg: Verlag Paul Parey; 1986.

[110] Sanetomo R, Hosaka K. A recombination-derived mitochondrial genome retained stoichiometrically only among *Solanum verrucosum* Schltdl. and Mexican polyploid wild potato species. Genet Resour Crop Evol. 2013;60:2391–404.

[111] Sanetomo R, Ono S, Hosaka K. Characterization of crossability in the crosses between *Solanum demissum* and *S. tuberosum*, and the F1 and BC1 progenies. Am J Potato Res. 2011;88:500–10.

[112] Sanetomo R, Habe I, Hosaka K. Sexual introgression of the late blight resistance gene *Rpi-blb3* from a Mexican wild diploid species *Solanum pinnatisectum* Dunal into potato varieties. Mol Breed. 2019;39:13.

[113] Shen W, Le S, Li Y, Hu F. SeqKit: a cross-platform and ultrafast toolkit for FASTA/Q file manipulation. PLoS ONE. 2016;11:e0163962. 27706213 10.1371/journal.pone.0163962 PMC5051824

[114] Simão FA, Waterhouse RM, Ioannidis P, Kriventseva EV, Zdobnov EM. BUSCO: assessing genome assembly and annotation completeness with single-copy orthologs. Bioinformatics. 2015;31:3210–2. 26059717 10.1093/bioinformatics/btv351

[115] Simon R, Fuentes AF, Spooner DM. Biogeographic implications of the striking discovery of a 4000 kilometer disjunct population of the wild potato *Solanum morelliforme* in South America. Syst Bot. 2011;36:1062–7.

[116] Song J, Bradeen JM, Naess SK, Raasch JA, Wielgus SM, Haberlach GT, et al. Gene *RB* cloned from *Solanum bulbocastanum* confers broad spectrum resistance to potato late blight. Proc Natl Acad Sci USA. 2003;100:9128–33. 12872003 10.1073/pnas.1533501100 PMC170883

[117] Spooner DM, Castillo-T R. Reexamination of series relationships of South American wild potatoes (Solanaceae: *Solanum* sect. *Petota*): evidence from chloroplast DNA restriction site variation. Am J Bot. 1997;84:671–85. 21708620

[118] Spooner DM, Knapp S. *Solanum stipuloideum* Rusby, the correct name for *Solanum circaeifolium* Bitter. Am J Potato Res. 2013;90:301–5.

[119] Spooner DM, Sytsma KJ. Reexamination of series relationships of Mexican and Central American wild potatoes (*Solanum* sect. *Petota*): evidence from chloroplast DNA restriction site variation. Syst Bot. 1992;17:432–48. 21708620

[120] Spooner DM, van den Berg RG, Bamberg JB. Examination of species boundaries of *Solanum* series *Demissa* and potentially related species in series *Acaulia* and series *Tuberosa* (sect. *Petota*). Syst Bot. 1995;20:295–314.

[121] Spooner DM, van den Berg RG, Rodríguez A, Bamberg J, Hijmans RJ, Cabrera SIL. Wild potatoes (*Solanum* section *Petota*; Solanaceae) of North and Central America. Systematic Botany Monographs 68. USA: The American Society of Plant Taxonomists, 2004.

[122] Spooner DM, Rodríguez F, Polgár Z, Ballard HE Jr, Jansky SH. Genomic origins of potato polyploids: GBSSI gene sequencing data. Crop Sci. 2008;48(S1):S27-36.

[123] Spooner DM, Ghislain M, Simon R, Jansky SH, Gavrilenko T. Systematics, diversity, genetics, and evolution of wild and cultivated potatoes. Bot Rev. 2014;80:283–383.

[124] Spooner DM, Alvarez N, Peralta IE, Clausen AM. Taxonomy of wild potatoes and their relatives in southern South America (*Solanum* sects. *Petota* and *Etuberosum*). Systematic Botany Monographs 100. USA: The American Society of Plant Taxonomists, 2016.

[125] Spooner DM, Ruess H, Arbizu CI, Rodriguez F, Solis-Lemus C. Greatly reduced phylogenetic structure in the cultivated potato clade (*Solanum* section *Petota* pro parte). Am J Bot. 2018;105:60–70. 29532930 10.1002/ajb2.1008

[126] Steuernagel B, Witek K, Krattinger SG, Ramirez-Gonzalez RH, Schoonbeek HJ, Yu G, et al. The NLR-annotator tool enables annotation of the intracellular immune receptor repertoire. Plant Physiol. 2020;183:468–82. 32184345 10.1104/pp.19.01273 PMC7271791

[127] Stiehler F, Steinborn M, Scholz S, Dey D, Weber APM, Denton AK. Helixer: cross-species gene annotation of large eukaryotic genomes using deep learning. Bioinformatics. 2021;36:5291–8. 33325516 10.1093/bioinformatics/btaa1044 PMC8016489

[128] Suyama M, Torrents D, Bork P. PAL2NAL: robust conversion of protein sequence alignments into the corresponding codon alignments. Nucleic Acids Res. 2006;34:W609–12. 16845082 10.1093/nar/gkl315 PMC1538804

[129] Swaminathan MS. Nature of polyploidy in some 48-chromosome species of the genus *Solanum*, section *Tuberarium*. Genetics. 1954;39:59–76. 17247468 10.1093/genetics/39.1.59 PMC1209637

[130] Swaminathan MS, Howard HW. The cytology and genetics of the potato (*Solanum tuberosum*) and related species. Bibliographia Genetica. 1953;16:1–192.

[131] Tang D, Jia Y, Zhang J, Li H, Cheng L, Wang P, et al. Genome evolution and diversity of wild and cultivated potatoes. Nature. 2022;606:535–41. 35676481 10.1038/s41586-022-04822-x PMC9200641

[132] van der Vossen E, Sikkema A, Hekkert BL, Gros J, Stevens P, Muskens M, et al. An ancient *R* gene from the wild potato species *Solanum bulbocastanum* confers broad-spectrum resistance to *Phytophthora infestans* in cultivated potato and tomato. Plant J. 2003;36:867–82. 14675451 10.1046/j.1365-313x.2003.01934.x

[133] van der Vossen EAG, Gros J, Sikkema A, Muskens M, Wouters D, Wolters P, et al. The *Rpi-blb2* gene from *Solanum bulbocastanum* is an *Mi-1* gene homolog conferring broad-spectrum late blight resistance in potato. Plant J. 2005;44:208–22. 16212601 10.1111/j.1365-313X.2005.02527.x

[134] Vossen JH, van Arkel G, Bergervoet M, Jo K-R, Jacobsen E, Visser RGF. The *Solanum demissum R8* late blight resistance gene is an *Sw-5* homologue that has been deployed worldwide in late blight resistant varieties. Theor Appl Genet. 2016;129:1785–96. 27314264 10.1007/s00122-016-2740-0 PMC4983296

[135] Watanabe K, Peloquin SJ. Cytological basis of 2*n* pollen formation in a wide range of 2*x*, 4*x*, and 6*x* taxa from tuber-bearing *Solanum* species. Genome. 1993;36:8–13. 18469967 10.1139/g93-002

[136] Xu X, Pan S, Cheng S, Zhang B, Mu D, Ni P, et al. Genome sequence and analysis of the tuber crop potato. Nature. 2011;475:189–95. 21743474 10.1038/nature10158

[137] Yamada T, Hosaka K, Nakagawa K, Kaide N, Misoo S, Kamijima O. Nuclear genome constitution and other characteristics of somatic hybrids between dihaploid *Solanum acaule* and tetraploid *S. tuberosum*. Euphytica. 1998;102:239–46.

[138] Yan LJ, Zhu ZG, Wang P, Fu CN, Guan XJ, Kear P, et al. Comparative analysis of 343 plastid genomes of *Solanum* section *Petota*: Insights into potato diversity, phylogeny, and species discrimination. J Syst Evol. 2023;61:599–612.

[139] Yang Z. PAML 4: phylogenetic analysis by maximum likelihood. Mol Biol Evol. 2007;24:1586–91. 17483113 10.1093/molbev/msm088

[140] Yang J, Liu D, Wang X, Ji C, Cheng F, Liu B, et al. The genome sequence of allopolyploid *Brassica juncea* and analysis of differential homoeolog gene expression influencing selection. Nat Genet. 2016;48:1225–32. 27595476 10.1038/ng.3657

[141] Yang X, Zhang L, Guo X, Xu J, Zhang K, Yang Y, et al. The gap-free potato genome assembly reveals large tandem gene clusters of agronomical importance in highly repeated genomic regions. Mol Plant. 2023;16:314–7. 36528795 10.1016/j.molp.2022.12.010

[142] Yin D, Ji C, Song Q, Zhang W, Zhang X, Zhao K, et al. Comparison of *Arachis monticola* with diploid and cultivated tetraploid genomes reveals asymmetric subgenome evolution and improvement of peanut. Adv Sci. 2020;7:1901672. 10.1002/advs.201901672 PMC7029647 32099754

[143] Yu G, Smith DK, Zhu H, Guan Y, Lam TTY. GGTREE: an R package for visualization and annotation of phylogenetic trees with their covariates and other associated data. Methods Ecol Evol. 2016;8:28–36.

[144] Zavallo D, Crescente JM, Gantuz M, Leone M, Vanzetti LS, Masuelli RW, et al. Genomic re-assessment of the transposable element landscape of the potato genome. Plant Cell Rep. 2020;39:1161–74. 32435866 10.1007/s00299-020-02554-8

[145] Zeng X, Yi Z, Zhang X, Du Y, Li Y, Zhou Z, et al. Chromosome-level scaffolding of haplotype-resolved assemblies using Hi-C data without reference genomes. Nat Plants. 2024;10:1184–200. 39103456 10.1038/s41477-024-01755-3

[146] Zhang RG, Li GY, Wang XL, Dainat J, Wang ZX, Ou S, et al. TEsorter: An accurate and fast method to classify LTR-retrotransposons in plant genomes. Hortic Res. 2022;9:uhac017. 35184178 10.1093/hr/uhac017 PMC9002660

[147] Zhang T, Hu Y, Jiang W, Fang L, Guan X, Chen J, et al. Sequencing of allotetraploid cotton (*Gossypium hirsutum* L. acc. TM-1) provides a resource for fiber improvement. Nat Biotechnol. 2015;33:531–7. 25893781 10.1038/nbt.3207

[148] Zhang Z, Zhang P, Ding Y, Wang Z, Ma Z, Gagnon E, et al. Ancient hybridization underlies tuberization and radiation of the potato lineage. Cell. 2025;188:1–17. 40749684 10.1016/j.cell.2025.06.034

[149] Zhou Y, Zhang Z, Bao Z, Li H, Lyu Y, Zan Y, et al. Graph pangenome captures missing heritability and empowers tomato breeding. Nature. 2022;606:527–34. 35676474 10.1038/s41586-022-04808-9 PMC9200638

