# Supplementary material for: Whole-genome sequencing revealed the genomic origin of Solanum demissum Lindl., a Mexican hexaploid wild potato species
Source: BMC Plant Biol. 2026 May 22;26:1208. doi: 10.1186/s12870-026-08972-2 (PMC13371551; doi:10.1186/s12870-026-08972-2)
Supplement: Supplementary file 1 — Supplementary Material 1: Fig. S1 Distributions of genes, transposable elements, HiFi-read coverages, heterozygous portions (heterozygous SNPs/kb), and subgenome-specific k-mers on the S. demissum chromosomes. Fig. S2 Chromosome-by-chromosome dS ratios among the S. demissum subgenomes (upper) and between each subgenome of S. demissum and the S. acaule subgenomes or S. verrucosum (lower). [file 12870_2026_8972_MOESM1_ESM.pdf]

Supplementary file 1

Article title:
Whole-genome sequencing revealed the genomic origin of *Solanum demissum* Lindl., a Mexican hexaploid wild potato species

Journal name: BMC Plant Biology

Author names: Awie J. Hosaka and Kazuyoshi Hosaka

Corresponding author: Kazuyoshi Hosaka
(Obihiro University of Agriculture and Veterinary; spudman@obihiro.ac.jp)

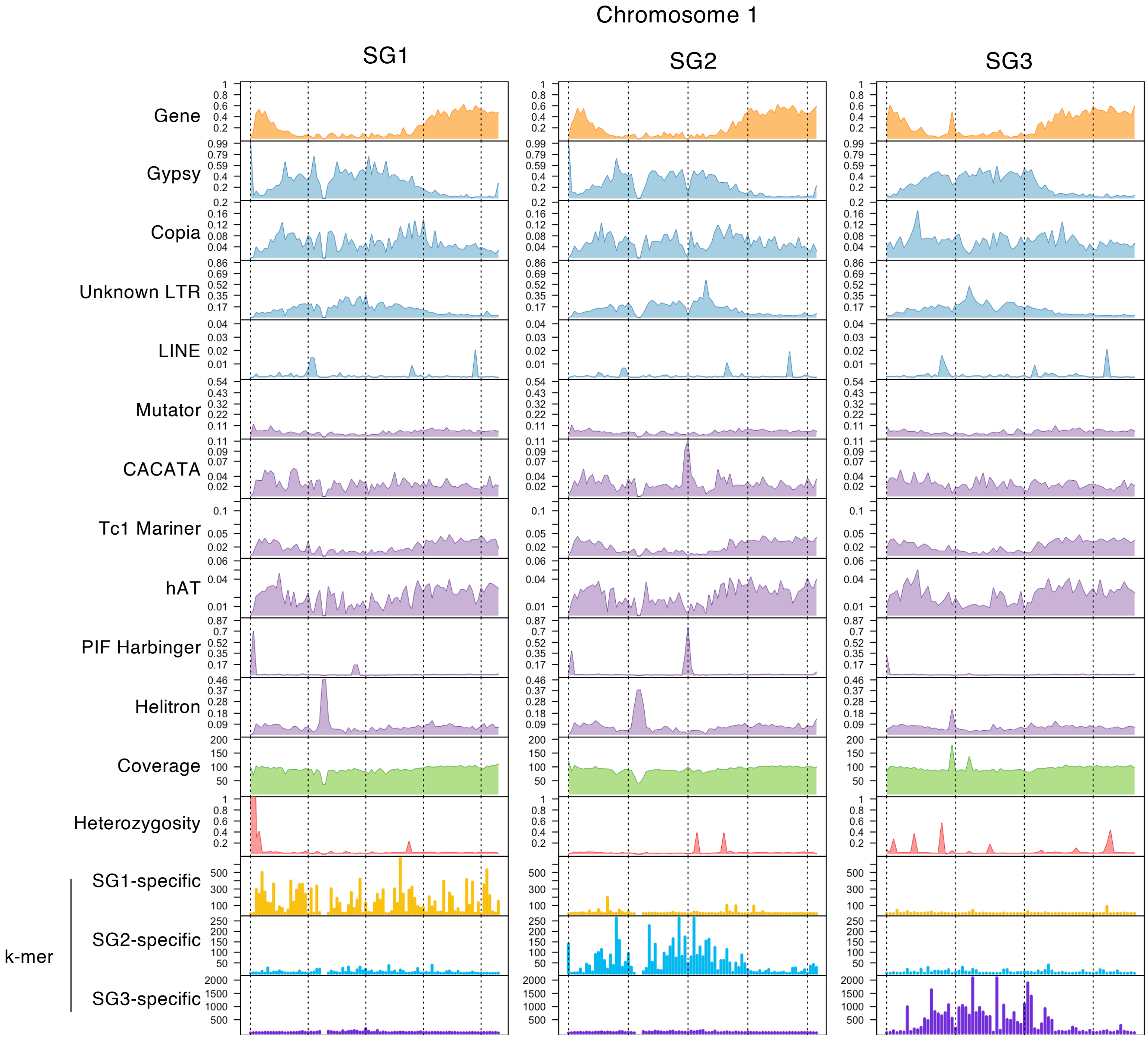

Fig. S1 Distributions of genes, transposable elements, HiFi-read coverages, heterozygous portions (heterozygous SNPs/kb), and subgenome-specific *k*-mers on the *S. demissum* chromosomes

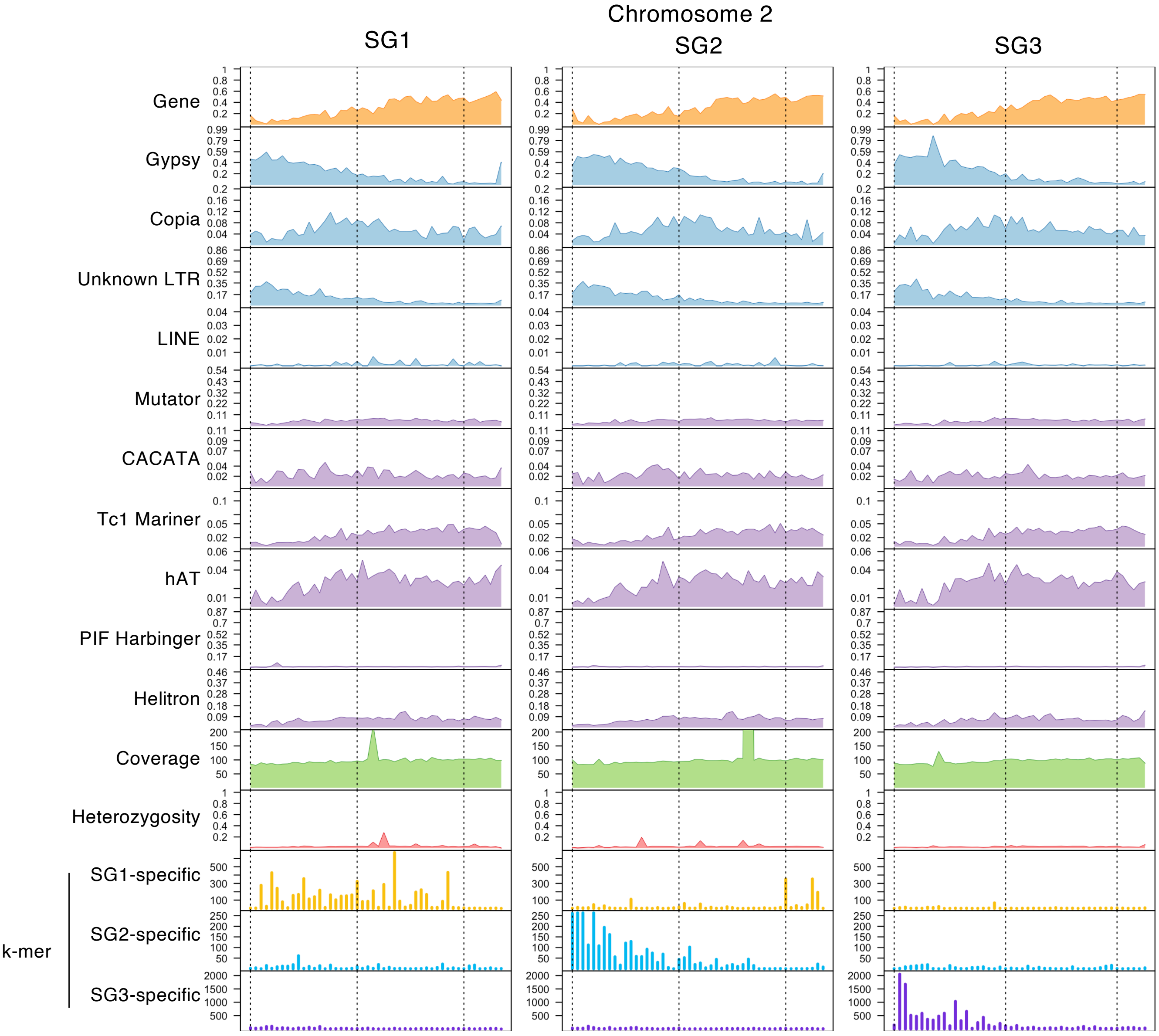

Fig. S1 (*continued*)

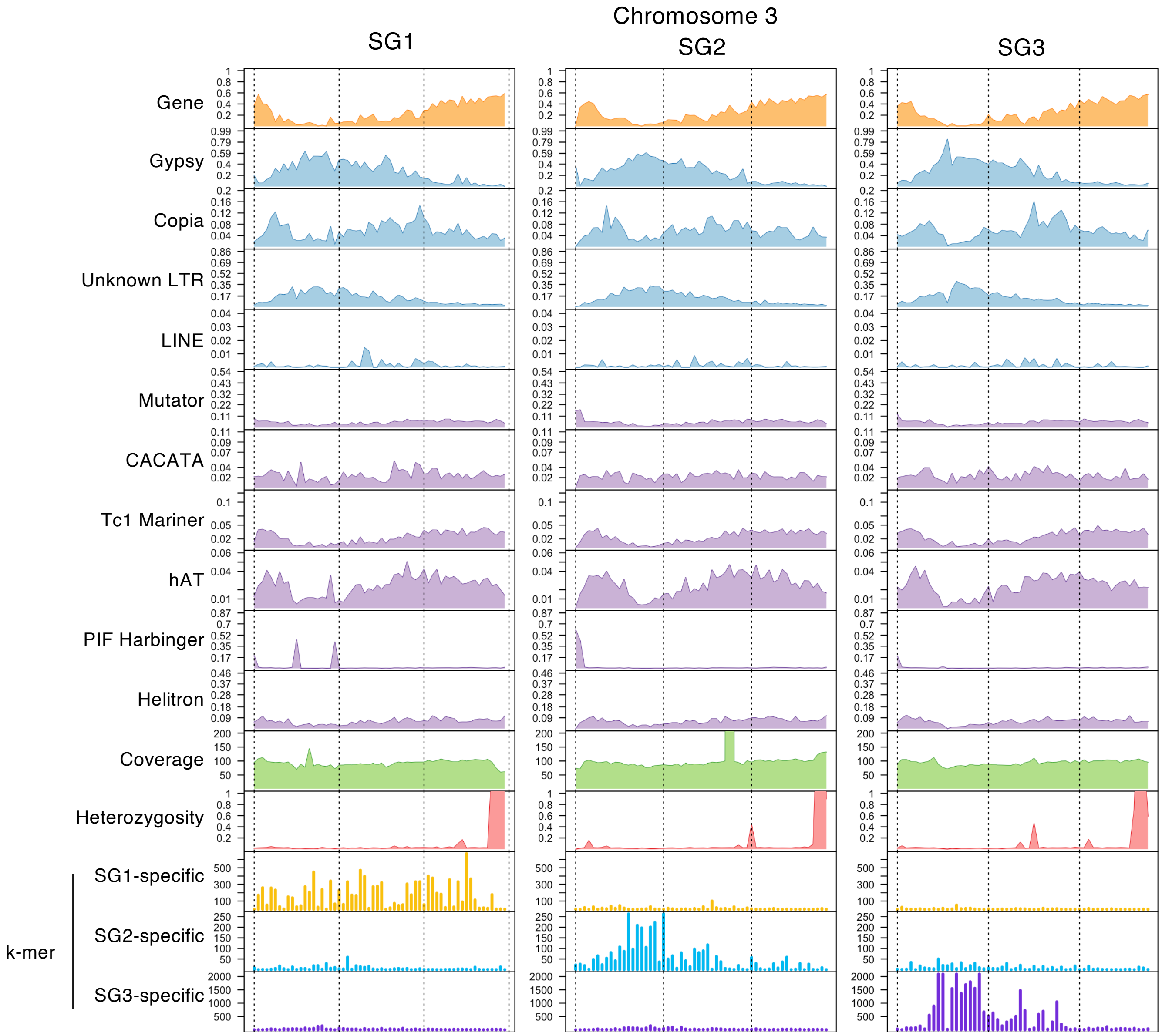

Fig. S1 (*continued*)

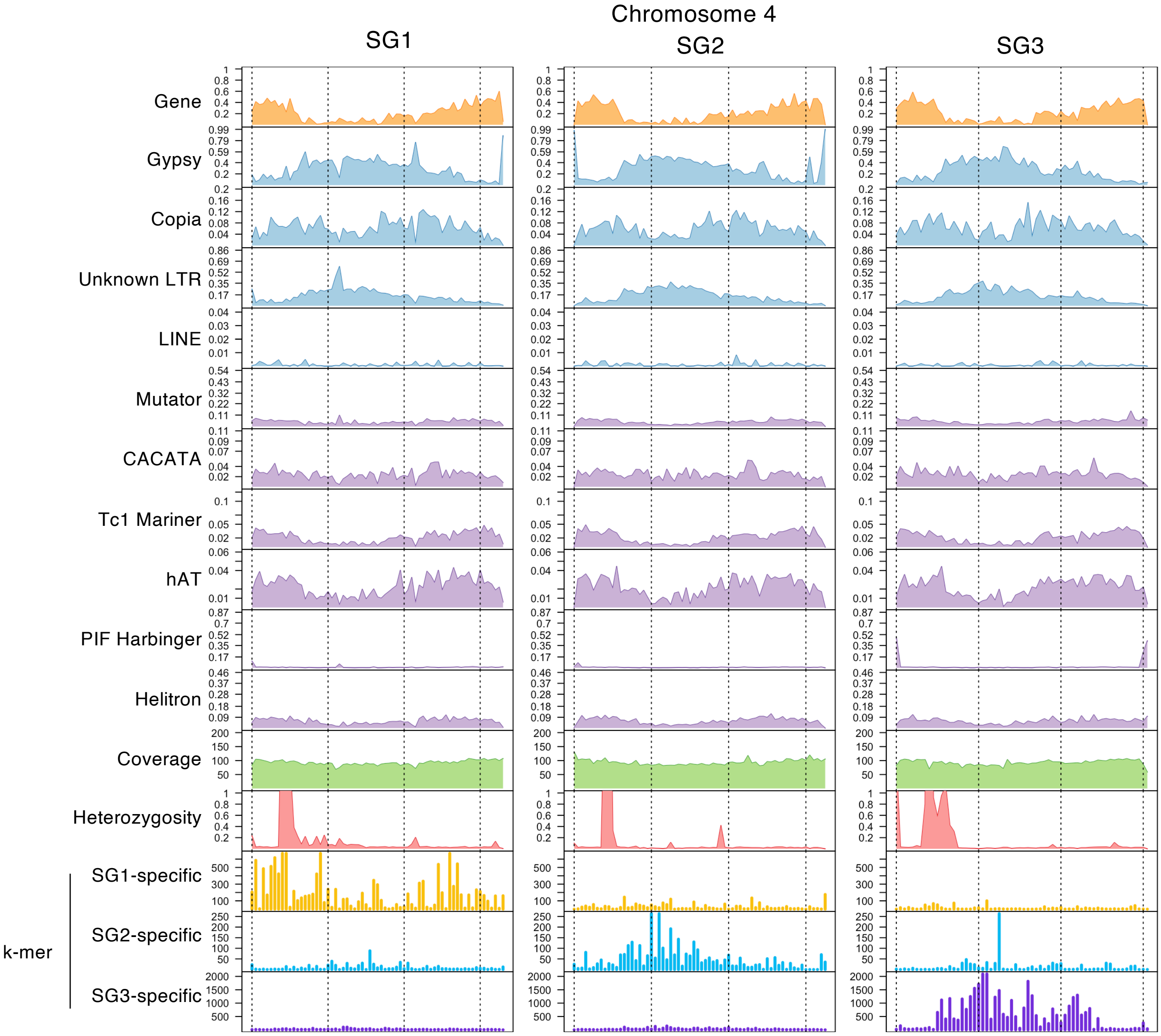

Fig. S1 (*continued*)

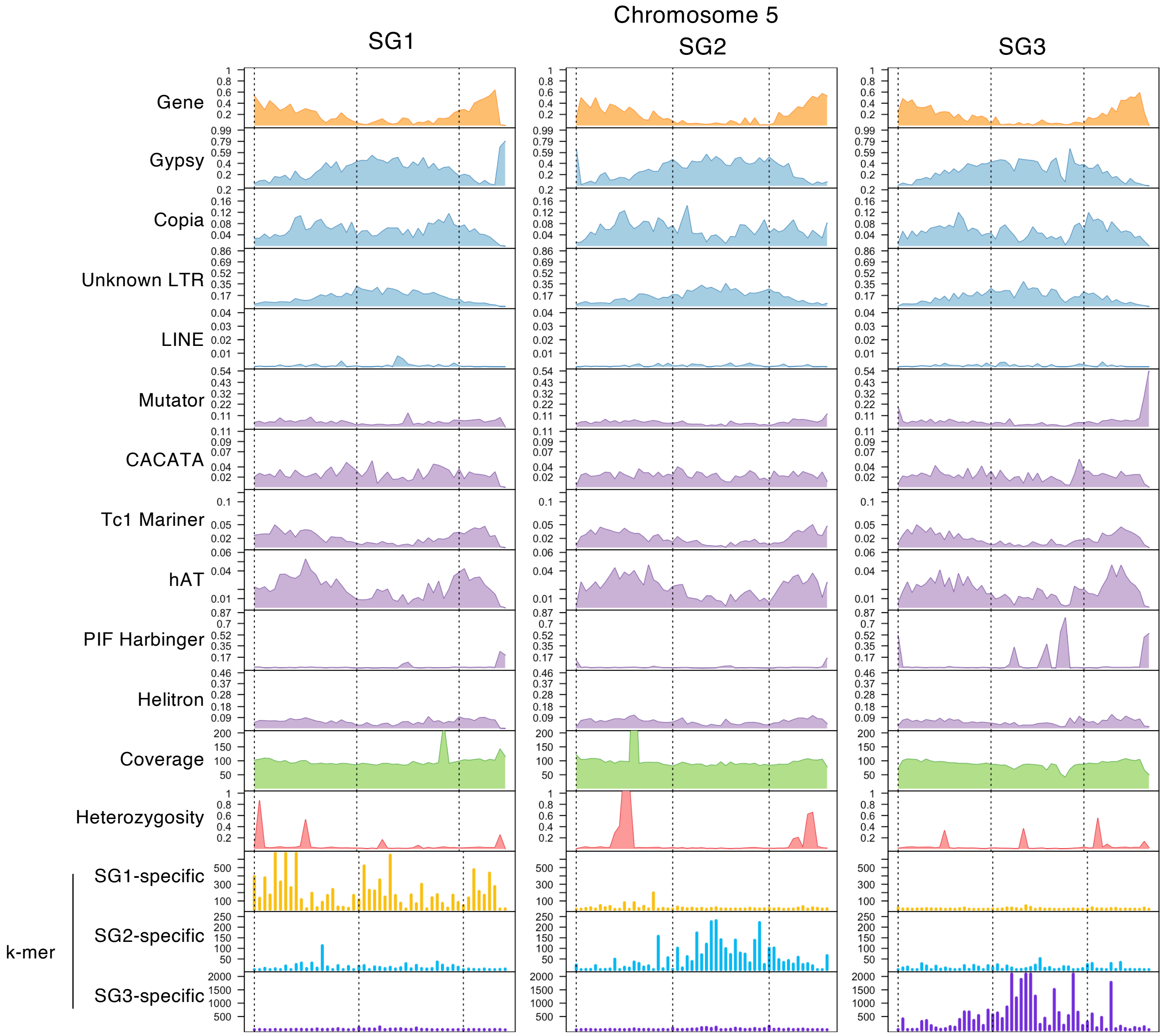

Fig. S1 (*continued*)

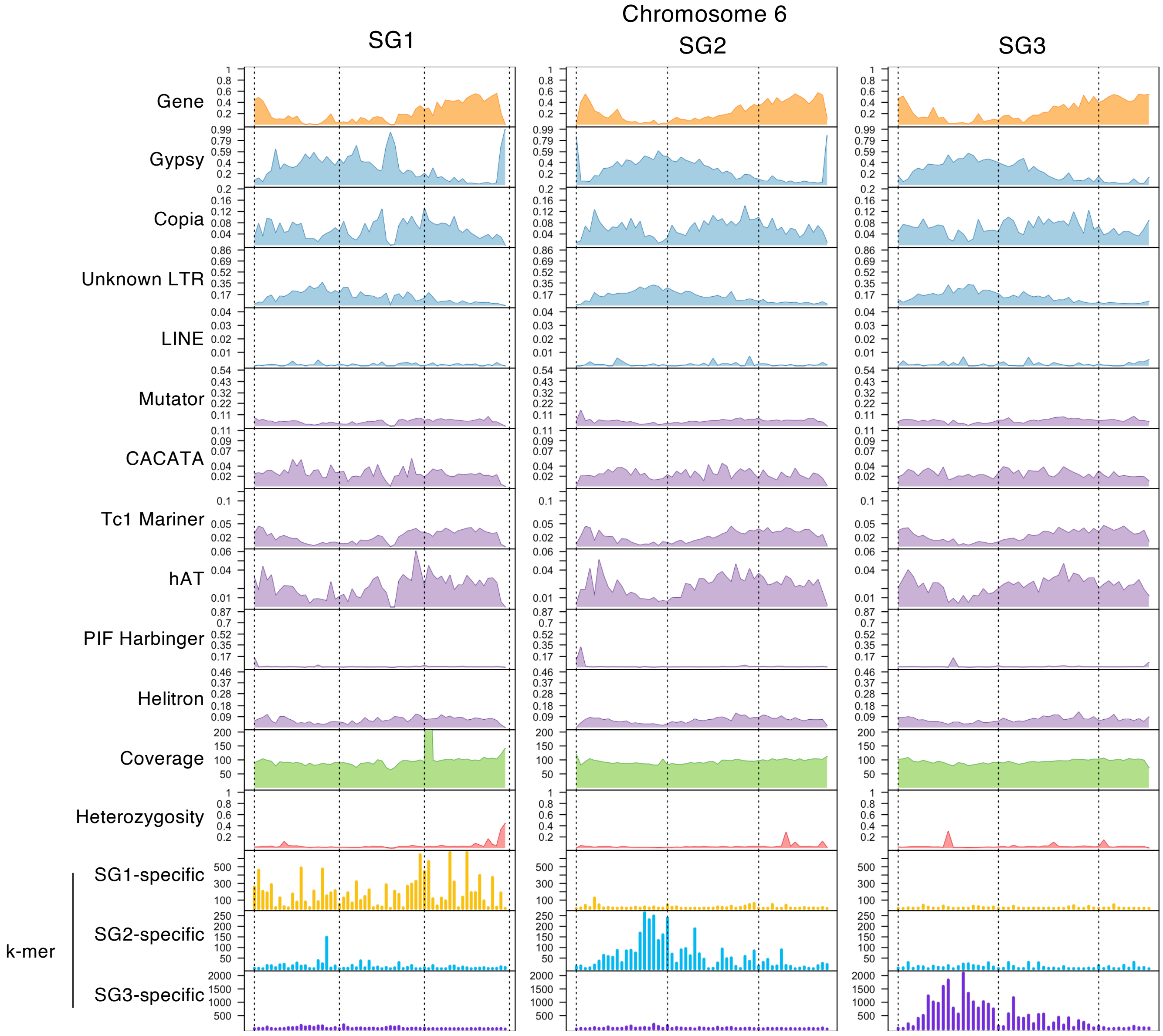

Fig. S1 (*continued*)

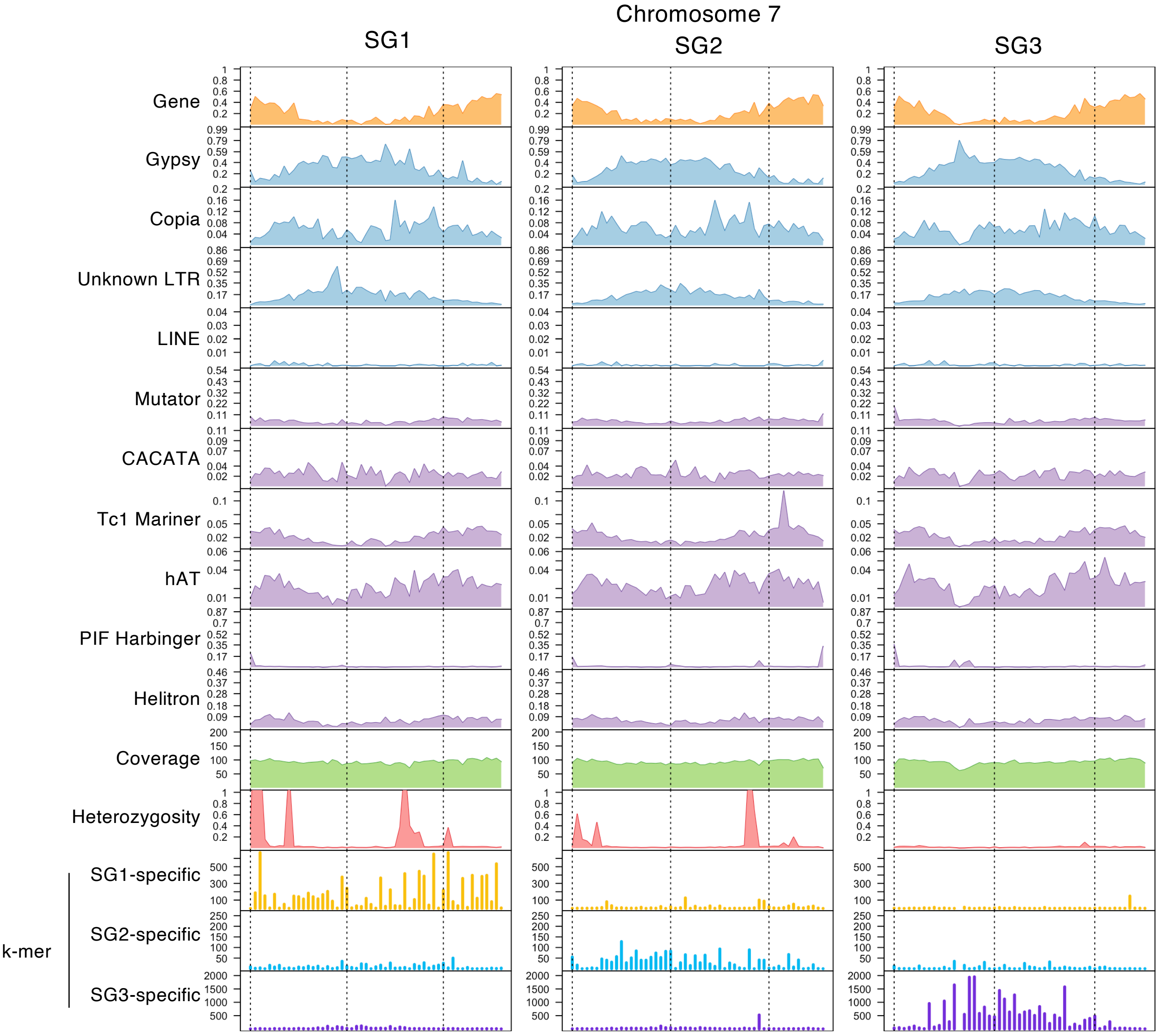

Fig. S1 (*continued*)

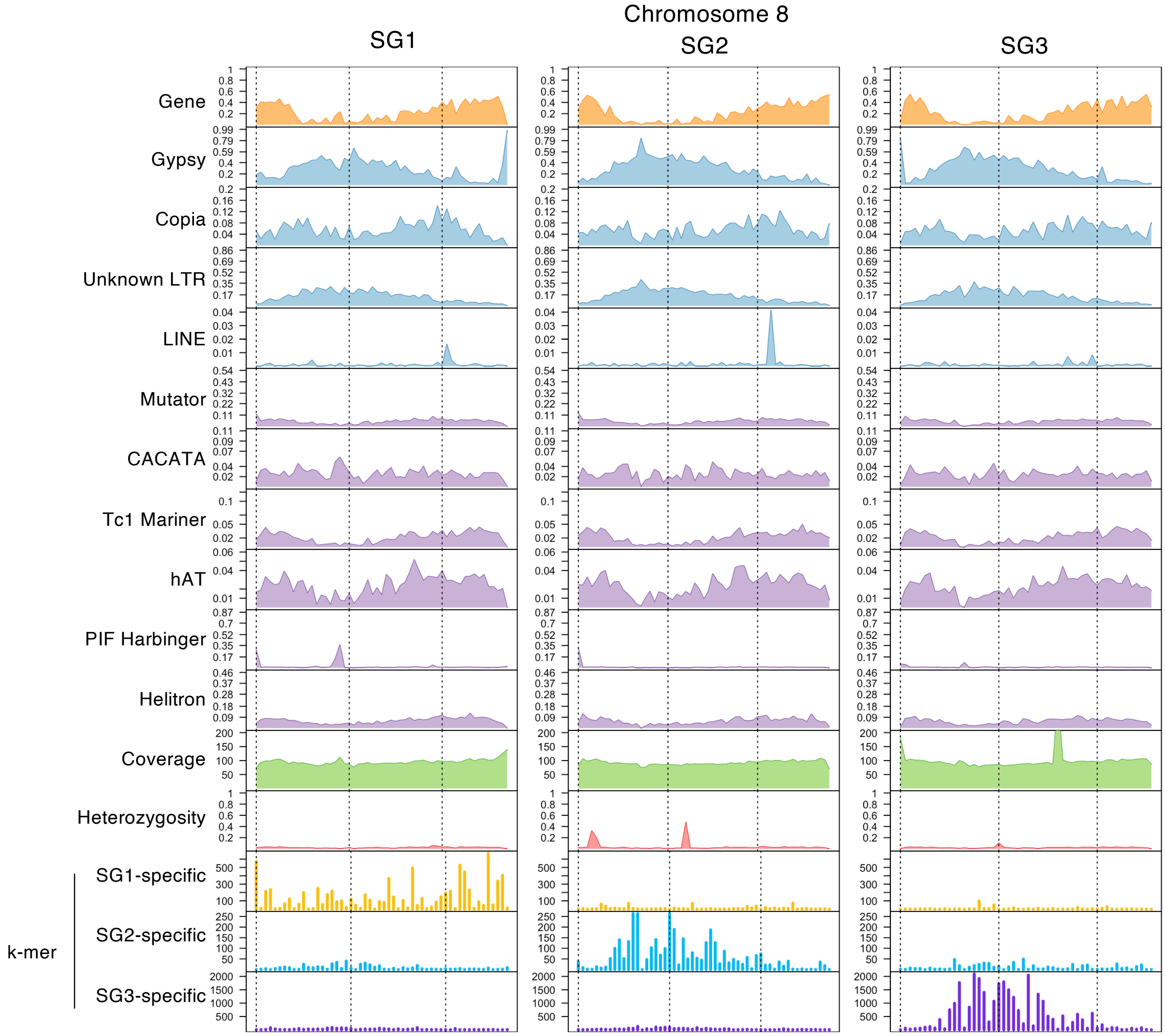

Fig. S1 (*continued*)

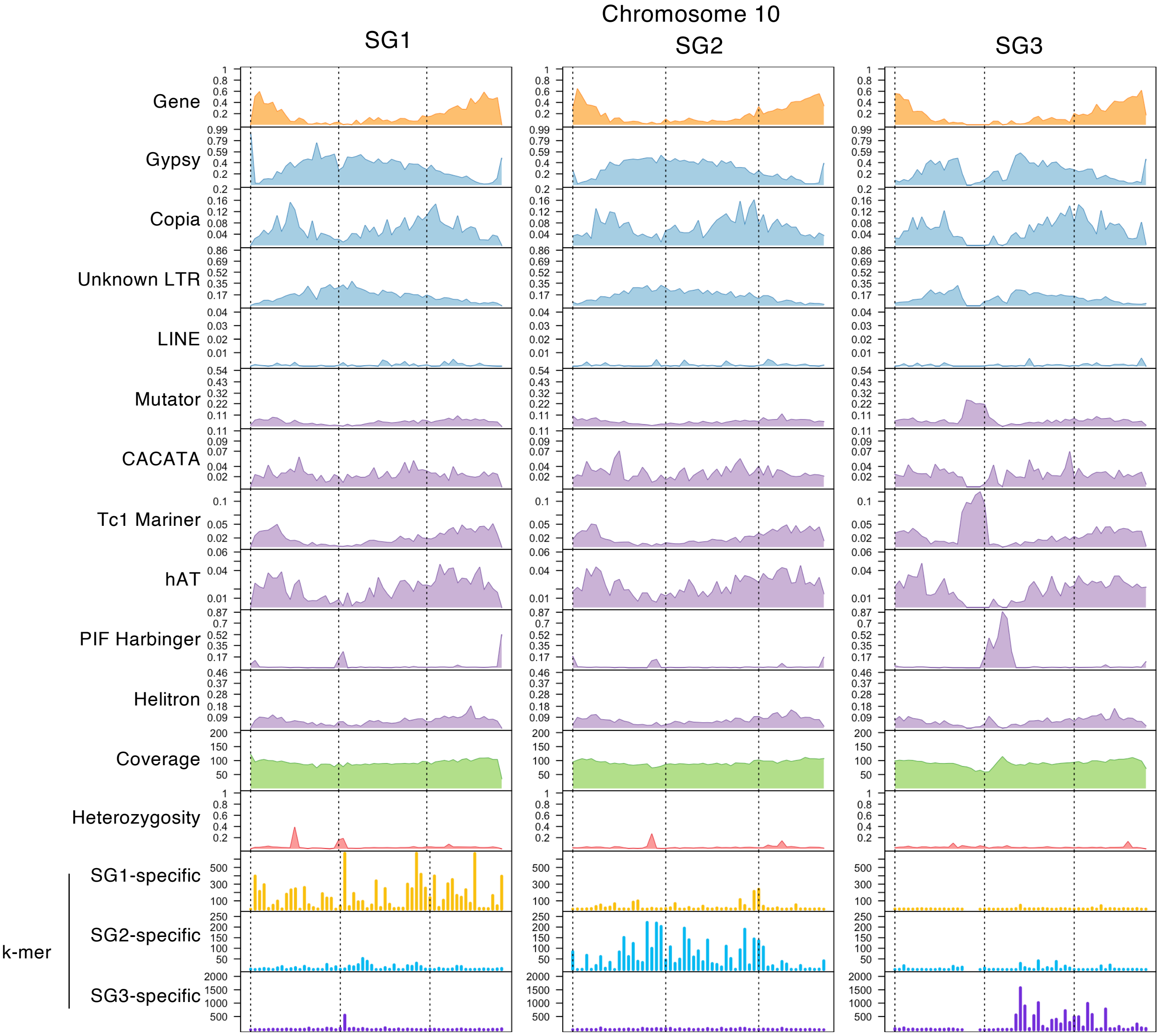

Fig. S1 (*continued*)

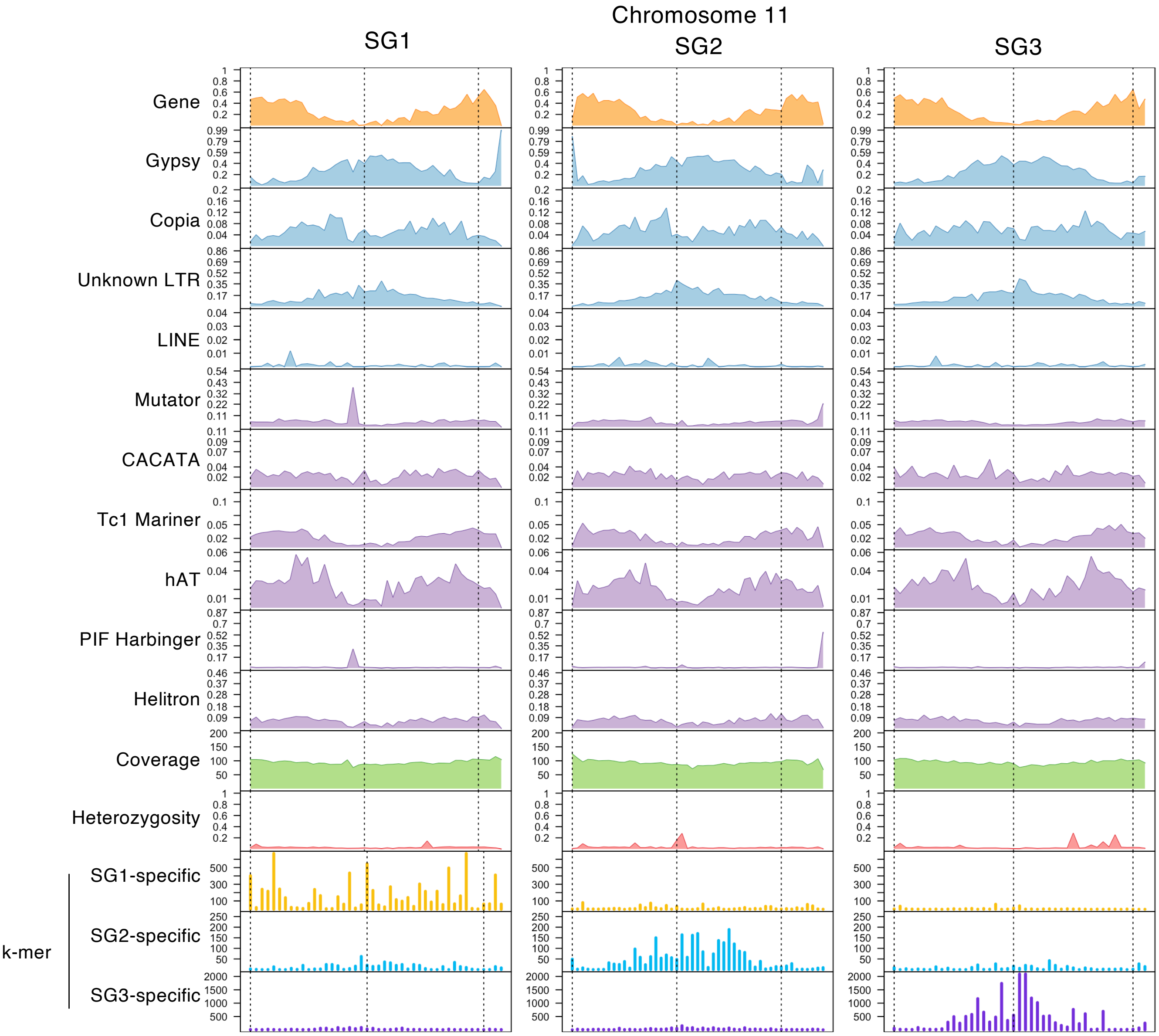

Fig. S1 (*continued*)

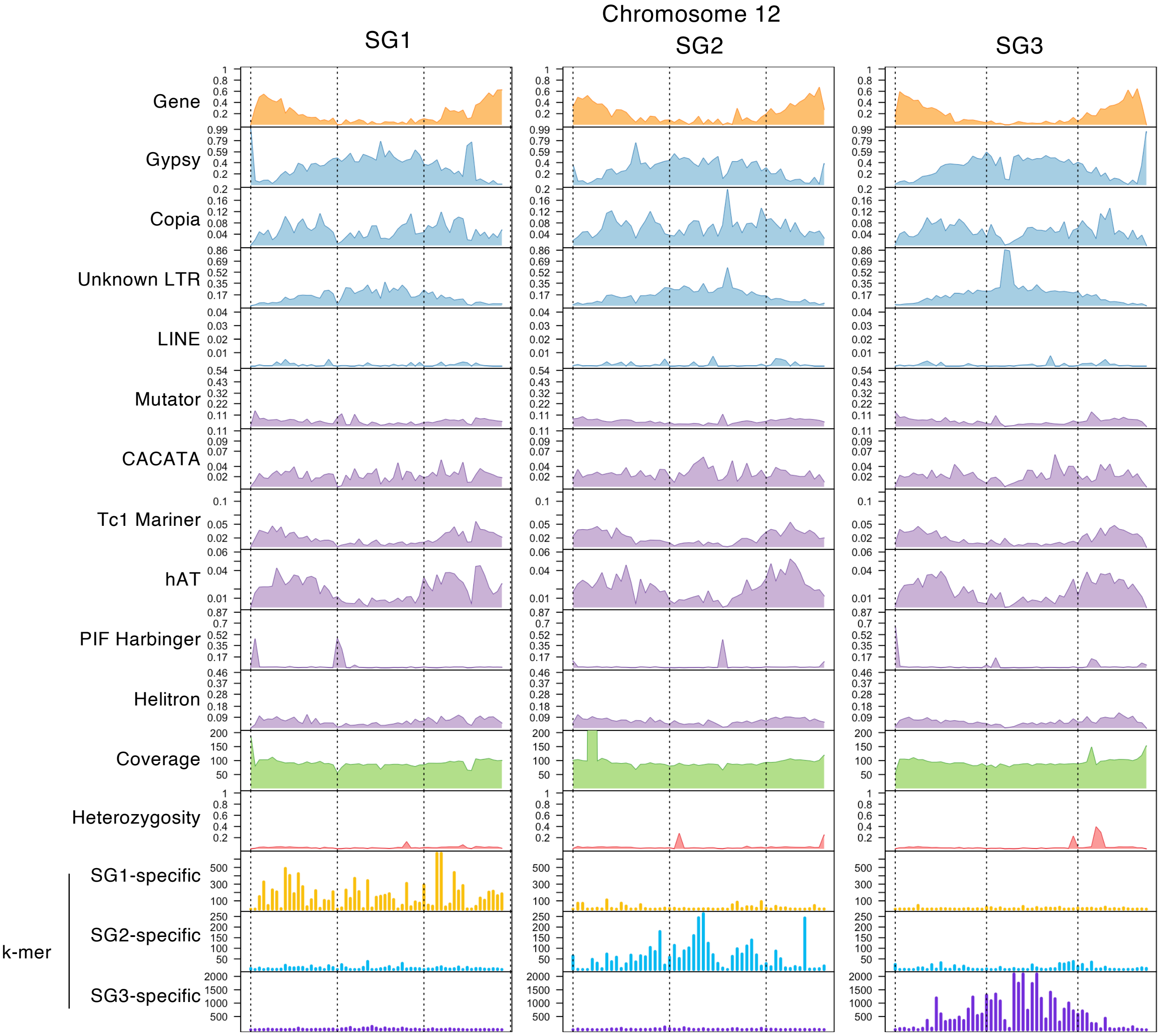

Fig. S1 (*continued*)

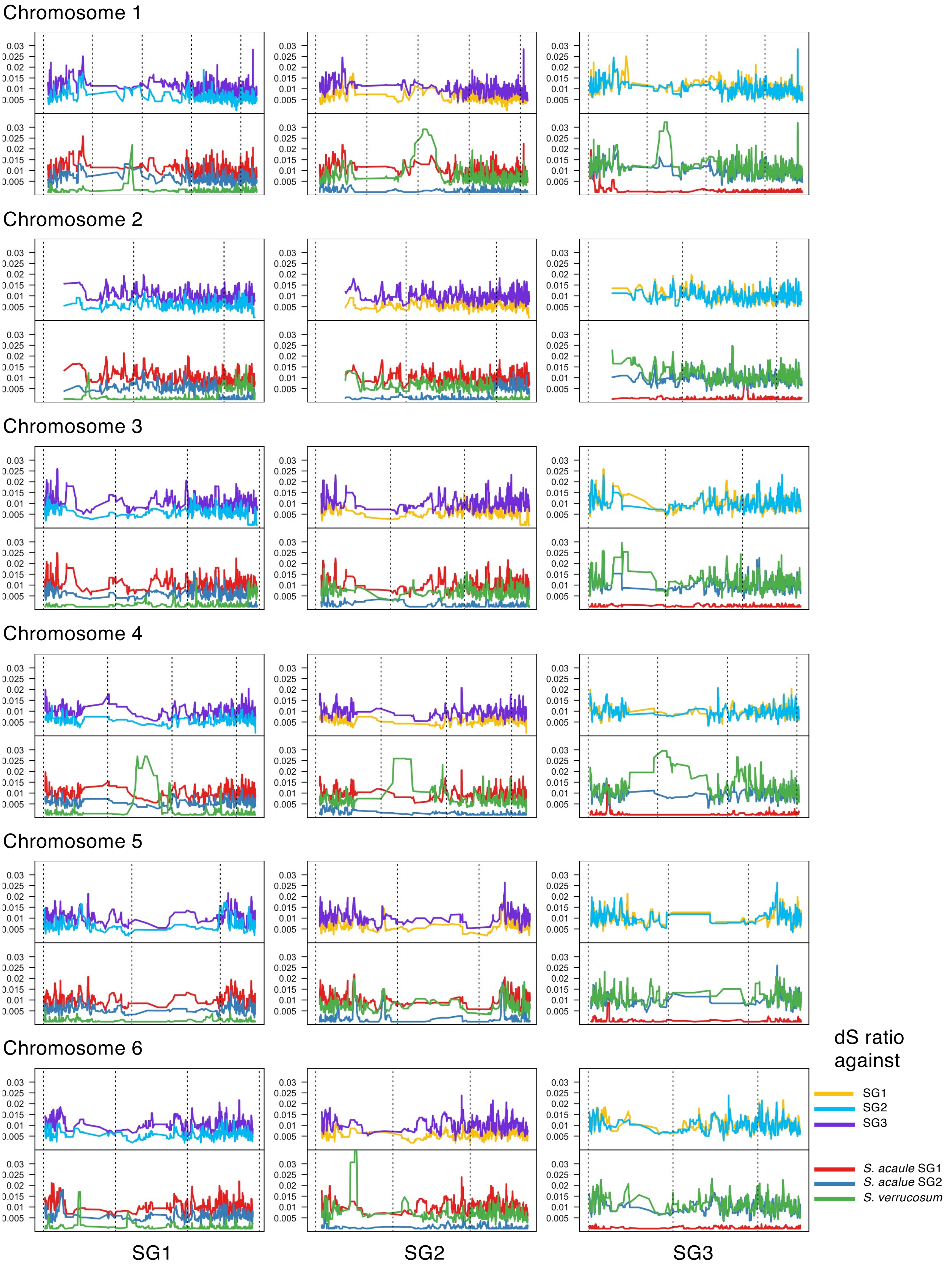

Fig. S2 Chromosome-by-chromosome dS ratios among the *S. demissum* subgenomes (upper) and between each subgenome of *S. demissum* and the *S. acaule* subgenomes or *S. verrucosum* (lower)

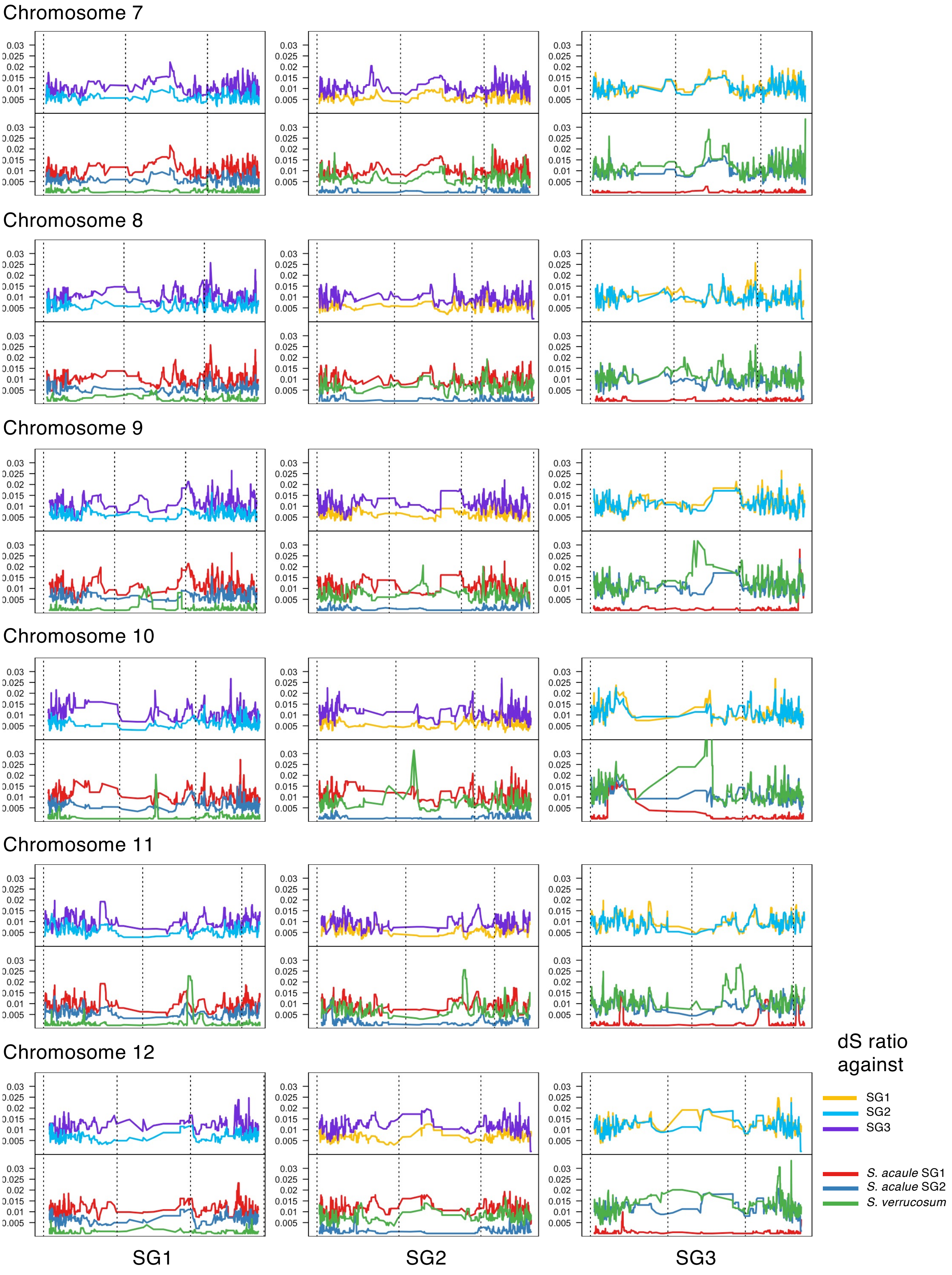

Fig. S2 (*continued*)
